# Individual differences moderate effects in an Unusual Disease paradigm: A psychophysical data collection lab approach and an online experiment

**DOI:** 10.3389/fpsyg.2023.1086699

**Published:** 2023-03-28

**Authors:** Marc Wyszynski, Adele Diederich

**Affiliations:** ^1^Department of Mathematics and Computer Science, University of Bremen, Bremen, Germany; ^2^Department of Psychology, University of Oldenburg, Oldenburg, Germany

**Keywords:** individual differences, framing effects, cognitive-style, risk-style, thinking-style, cognitive-experiential self-theory, framing susceptibility, frame-inconsistent choice

## Abstract

We report two studies investigating individual intuitive-deliberative cognitive-styles and risk-styles as moderators of the framing effect in Tversky and Kahneman's famous Unusual Disease problem setting. We examined framing effects in two ways: counting the number of frame-inconsistent choices and comparing the proportions of risky choices depending on gain-loss framing. Moreover, in addition to gain-loss frames, we systematically varied the number of affected people, probabilities of surviving/dying, type of disease, and response deadlines. Study 1 used a psychophysical data collection approach and a sample of 43 undergraduate students, each performing 480 trials. Study 2 was an online study incorporating psychophysical elements in a social science approach using a larger and more heterogeneous sample, i.e., 262 participants performed 80 trials each. In both studies, the effect of framing on risky choice proportions was moderated by risk-styles. Cognitive-styles measured on different scales moderated the framing effect only in study 2. The effects of disease type, probability of surviving/dying, and number of affected people on risky choice frequencies were also affected by cognitive-styles and risk-styles but different for both studies and to different extents. We found no relationship between the number of frame-inconsistent choices and cognitive-styles or risk-styles, respectively.

## 1. Introduction

Since Tversky and Kahneman (1981) seminal paper on framing, numerous studies have shown that decisions under risk are often influenced by the way the decision problem is presented. This phenomenon, known as framing effect, violates the normative principle of description invariance; that is, a decision must not depend on the way how it is presented. Presumably, the most famous and most applied example for framing risky choice alternatives is Tversky and Kahneman (1981) Unusual Disease Problem.^1^ The problem describes two programs to combat a hypothetical disease that is expected to kill 600 people in either a positive or a negative frame. In the positive (negative) frame, 200 people can be saved (400 will die) for sure with program A (C), or 600 people will be saved (will die) with a probability of 1/3 (2/3) with program B (D). Most of the participants chose program A in the positive frame and program D in the negative frame. The framing effect in Unusual Disease Problems has repeatedly been demonstrated by more than 40 studies (see e.g., Kühberger, 1998; Levin et al., 1998; Kühberger et al., 1999; Piñon and Gambara, 2005; Steiger and Kühberger, 2018, for meta-analytic reviews).

The framing effect is typically accounted for by prospect theory (Kahneman and Tversky, 1979), but more recently the notion of dual processes have been brought into play. According to this approach, framing effects result from the interplay of two different systems of reasoning. One system, generically called “System 1,” includes fast and intuitive processes, whereas the other system, often called “System 2,” is described in terms of slow and deliberative processing (see e.g., Chaiken and Trope, 1999; Stanovich and West, 2000; Evans, 2008; Mukherjee, 2010; Guo et al., 2017; Roberts et al., 2021). Framing effects mainly emerge in the fast and intuitive System 1, and they tend to disappear when the slow and deliberative System 2 is engaged (see e.g., Sloman, 1996; Kahneman and Frederick, 2002, 2005). Empirical findings support these hypotheses: stronger framing effects are observed when participants are put under time pressure (Guo et al., 2017; Diederich et al., 2018, 2020; Wyszynski et al., 2020; Roberts et al., 2021; Wyszynski and Diederich, 2022), and weaker framing effects occur when people are forced to use deliberative reasoning (Miller and Fagley, 1991; Takemura, 1994; Sieck and Yates, 1997; Almashat et al., 2008).

If experimental manipulations inducing intuitive or deliberative processing can affect the strength of the framing effect then it is possible that the decision-makers individual style of processing information intuitively or deliberately may also moderate framing effects (Stanovich, 1999; Evans, 2008; Mandel and Kapler, 2018).

The cognitive-experiential self-theory (CEST; Epstein, 1994) originally introduced as a global theory of personality (Epstein, 1973) assumes a rational and an experiential system. In both systems people have constructs about the self and the world, referred to as schemata (rational) and beliefs (experiential). The experiential system has been linked to heuristics. Furthermore, CEST assumes “important individual differences in the relative degree and effectiveness with which individuals use the two modes of information processing” (Epstein, 1994, p. 719). Several scales based on CEST to measure those differences have been constructed. For instance, Epstein et al. (1996) developed the Rational-Experiential Inventory (REI) that consists of a modified version of the Need for Cognition scale (NFC, Cacioppo and Petty, 1982) and the Faith in Intuition scale (FI, Epstein et al., 1996). NFC is a measure of deliberative-rational cognitive-style. In particular, it “refers to an individual's tendency to engage in and enjoy effortful cognitive endeavors” (Cacioppo et al., 1984, p. 306). Decision makers with a high NFC score are expected to be less susceptible to the effect of framing. A person's FI reflects its intuitive-experiential processing (Epstein et al., 1996), which is characterized by a rapid, holistic, and emotional cognitive-style. Decision-makers who score high in FI are expected to produce more framing effects than those with lower FI scores.

Another concept of deliberative thinking-style is Actively Open-Minded Thinking (AOT, Baron, 1993). AOT style is characterized by the tendencies “to weight new evidence against a favored belief, to spend sufficient time on a problem before giving up, and to consider carefully the opinions of others in forming one's own” (Haran et al., 2013, p. 189). Higher AOT is associated with better decision-making performance, i.e., producing fewer framing effects. The Stimulating-Instrumental Risk Inventory (SIRI, Zaleskiewicz, 2001) measures individual risk-styles based on rational-experiential processing modes. Stimulating risk is associated with experiential risk-style and the enjoyment of risk. It may lead to faster, less analytical, and more heuristic decisions. Instrumental risk-taking relates to the rational system. High instrumental risk-takers are expected to analyze the characteristics and values of a risky choice carefully and, therefore, produce fewer framing effects.

Individual differences have been linked to framing susceptibility but the results are mixed. Some studies indicated that individual differences in intuitive-deliberative cognitive-styles and risk-styles moderate framing effects in the expected way (NFC: e.g., LeBoeuf and Shafir, 2003; Simon et al., 2004; Björklund and Bäckström, 2008; Peng et al., 2019; AOT: e.g., West et al., 2008; Erceg et al., 2022; Rachev et al., 2022), and other research, however, failed to identify a significant relationship (NFC: e.g., Corbin, 2015; Fatmawati, 2015; Stark et al., 2017; Mandel and Kapler, 2018; FI: e.g., Levin et al., 2002; Shiloh et al., 2002; Björklund and Bäckström, 2008; Stark et al., 2017; AOT: e.g., Erceg et al., 2022, study 2; Mandel and Kapler, 2018; SIRI: e.g., Mahoney et al., 2011).

These studies, however, differ substantially in the framing effect interpretations, characteristics of the decision problems presented in the experiments, sample compositions, and study designs, which may explain the discrepancy of their results. In particular, several interpretations of framing effects have been used. Peng et al. (2019) and Rachev et al. (2022) used the “resistance to framing” component of the Adult Decision Making Competence (ADMC) scale (Bruine de Bruin et al., 2007). This involves one unusual disease-like decision problem framed as gain or loss. West et al. (2008) and Mandel and Kapler (2018) counted the frame-(in)consistent choices participants made in one risky decision problem. Mandel and Kapler (2018) counted a “frame-consistent” choice if participants chose the sure option in the gain frame, and the risky option in the loss frame in a between-subjects design. West et al. (2008) counted a “frame-inconsistent” choice when participants chose the sure option in one frame and the risky option in the other frame in a within-subjects study. Other studies evaluated individual differences in proportions of choosing the sure and the risky option depending on the framing of the decision problem (e.g., Shiloh et al., 2002; Simon et al., 2004). Different framing interpretations may account for differences in strength of the framing effect and its correlation with psychometric instruments.

Furthermore, certain characteristics describing the decision problem, such as probabilities, magnitude of outcome, problem domain, and different time limits for making a choice, have been shown to influence risky choice additionally to framing (see Kühberger et al., 1999; Mahoney et al., 2011; Diederich et al., 2018, for overviews). Only a few studies investigating the impact of cognitive-styles and/or risk-styles on risky choice framing effects varied one or more problem-describing characteristics in their experiments (e.g., Mahoney et al., 2011; Corbin, 2015). None of them report any results about the relationship between problem-describing characteristics and individual differences in risky choices.

Whether or not cognitive-style and/or risk-style moderate the framing effect may further depend on the sample composition. In previous studies, many samples were composed of undergraduate or graduate university students. Using student samples could be seen as a kind of pre-selection or screening because student samples are more homogeneous and may provide a limited range of psychometric scores measured using a particular instrument as compared to a community or online sample (see, e.g., Peterson, 2001).

Finally, the study design may not have been optimal in several cases. While some studies varied the framing manipulation within participants (e.g., Levin et al., 2002; Mahoney et al., 2011; Peng et al., 2019; Erceg et al., 2022; Rachev et al., 2022), other studies relied on between-subjects designs where a particular decision problem is described by different frames and each participant responds to only one of these frames (Shiloh et al., 2002; Simon et al., 2004; Björklund and Bäckström, 2008; Fatmawati, 2015; Stark et al., 2017; Mandel and Kapler, 2018). However, several researchers pointed out that a within-subjects design is more appropriate when investigating framing effects on the individual level (Frisch, 1993; Baron, 2010; Appelt et al., 2011; Mahoney et al., 2011; Aczel et al., 2018). It allows analyzing an individual's susceptibility to framing effects based on certain individual characteristics such as cognitive-styles and risk-styles.

A key challenge in investigating framing effects using within-subjects designs is the transparency of framing manipulation. Once participants notice the similarity between frames, they may tend to give the same response in both frames (Aczel et al., 2018). The common way of dealing with this problem is adding intervening steps between the two frames, for instance, by inserting a temporal break (e.g., Levin et al., 2002; Parker and Fischhoff, 2005), inserting filling questions (e.g., Stanovich and West, 1998; LeBoeuf and Shafir, 2003; Li and Liu, 2008), or masking the frames by presenting different problems in random order (e.g., Frisch, 1993). However, framing effect strengths are often smaller in within-subjects studies than in between-subjects designs (Piñon and Gambara, 2005; Aczel et al., 2018). This difference is still commonly explained by the higher transparency of manipulations in within-subjects designs (Kahneman and Frederick, 2005).

To overcome these problems, Mahoney et al. (2011) introduced an alternative approach using a within-subjects design: The Unusual Disease Problem varied with respect to the specific disease, the number of affected people, and probabilities of surviving/dying to create five unique choice problems, each framed as gain and loss. They found strong framing effects. However, the results did not support their hypothesis that individual cognitive-styles and risk-styles moderate the framing effect.

To shed some more light on the mixed results in previous research, we have the following goals. First, we seek to extend the within-subjects study of Mahoney et al. (2011) by using a psychophysical data collection approach in experiment 1. That is, instead of presenting few trials to many participants as in a typical social science approach, here fewer participants perform many more trials. This method had successfully been used in other framing studies (Guo et al., 2017; Diederich et al., 2020; Roberts et al., 2021; Wyszynski and Diederich, 2022). Second, we include two different interpretations of the framing effect: a narrow interpretation, i.e., comparing the number of frame-inconsistent choices between participants; and a wide one, i.e., comparing the proportions of risky choices made by the participants in the two frames. Third, we include variables defining the choice problems as explanatory variables. Fourth, we seek to replicate the results of our first experiment using an online-sample to overcome a potential homogeneity issue of student samples. For the online experiment, we incorporate the psychophysical approach from experiment 1 into a social science approach requiring a larger sample size in favor of fewer trials per participant. The combined design has three advantages for our study: (1) a larger and more heterogeneous sample provides a broader range of psychometric cognitive-style and risk-style scores; (2) the correlations between frame-inconsistent choices and scores measured with psychometric instruments are expected to be more stable in larger samples (Schönbrodt and Perugini, 2013); and (3) due to the smaller number of trials, participants are less likely to drop out during the online session.

## 2. Experiment 1

The first experiment was done in a lab using a quasi psychophysical approach. Participants were asked to choose either the sure or the risky (gamble) option in a series of Unusual Disease Problems. Choice and response time data are based on Diederich et al. (2018) who investigated several determinants of risky decision making utilizing a sample of students receiving monetary compensation. Similar to Mahoney et al. (2011), the study used three different diseases embedded into two frames. Details on the number of affected people, probabilities, and response deadline variations are described in the following. For the current study, we elected scores on different psychometric instruments to examine the influence of cognitive-style and risk-style on choice behavior.

### 2.1. Materials

In addition to the framing manipulations, i.e., presenting each trial in a gain and a loss frame, Diederich et al. (2018) included four variables (characteristics) describing the choice problem: outcomes, probabilities of surviving/dying, problem domain, and time limits.

**Outcomes**: The outcomes of the decision were described as the number of people affected by a certain disease. Diederich et al. (2018) defined two major categories for the number of affected people, called *Scope* here. Category Small included the values 20, 40, 60, and 80. To minimize a possible impact of prominent numbers on risky choice, each value was flanked by ±1 resulting in four triplets of values (19, 20, 21; 39, 40, 41; 59, 60, 61; 79, 80, 81). For category Large, these numbers were multiplied by 100.

**Probabilities**: The probability indicated for a particular choice problem describes the affected peoples' chance of survival/death. Probabilities of surviving/dying varied on four levels. The particular values were 0.3, 0.4, 0.6, and 0.7.

**Problem domain**: The problem domain was varied by including three different versions of the Unusual Disease Problem (scenarios). For the control condition of the disease variable, the scenario described an outbreak of an unusual infectious disease (category: Infectious). The other two Unusual Disease Problem scenarios were about a new agent to treat leukemia (category: Leukemia) and a new agent to treat AIDS (category: AIDS). The full texts of the disease scenarios can be found in the Supplementary material.

**Time limits**: Two response deadlines were included. A short time limit of 1 s and a longer time limit of 3 s.

For a given Scope, the twelve numbers of affected people were paired with the probabilities to 48 combinations (12 × 4) per frame resulting in 96 individual test trials. The sure option for each trial was created to match the expected value of the gamble option. In addition, 24 catch trials (12 per frame) were constructed to assess accuracy and engagement in the task. The catch trials had two non-equivalent choice options. One option had a significantly larger expected value than the other option. For the catch trials, a probability of 0.9 (0.1) for risky options was paired with the expected value of the number of affected people multiplied by 0.1 (0.9). The sure option was preferable to the risky option for 12 catch trials (6 per frame), and vice versa for the other 12 catch trials (for details see Diederich et al., 2018). Altogether, 96 test trials plus 24 catch trials make 120 trials presented in one block. Furthermore, a block of trials was embedded in one disease category and one level of time limits.

### 2.2. Measures

We measured cognitive-styles with two different inventories. First, similarly to Mahoney et al. (2011), we used the 40-items Rational-Experiential Inventory (REI-40), with the rational-analytic (RA) and the experiential-intuitive (EX) sub-scales (Pacini and Epstein, 1999). RA thinking is equivalent to the concept of Need for Cognition (NFC). It is measured by an adapted version of the original NFC instrument (Cacioppo and Petty, 1982). EX thinking is basically equivalent to the Faith in Intuition (FI) concept (Epstein et al., 1996). Participants rated all items on a 5-point Likert scale that ranged from 1 (“definitely not true of myself”) to 5 (“definitely true of myself”). We observed a reliability of RA and EX of α = 0.86 and α = 0.84, respectively.

Second, we used the 7-item short form of the Actively Open-Minded Thinking (AOT-7) scale as used in Haran et al. (2013), who investigated the role of AOT in the acquisition, accuracy, and calibration of information. Participants rated all items on a 7-point Likert scale from 1 (“completely disagree”) to 7 (“completely agree”). In the current study, the reliability of the AOT scale was α = 0.7.

We measured risk-styles with the Stimulating-Instrumental Risk Inventory (SIRI; Zaleskiewicz, 2001), which is composed of two sub-scales, the stimulating-risk sub-scale (ST) and the instrumental-risk sub-scale (IN). Participants have to self-assess their attitudes to 17 statements (10 ST, 7 IN) using a 5-point Likert scale from 1 (“does not describe me at all”) to 5 (“describes me very well”). In the current study, the reliability was α = 0.74 for the ST scale and α = 0.58 for the IN scale.

The questionnaires, as they were used in this study, are found in the Supplementary material.

### 2.3. Design and procedure

The study had a mixed design. Three diseases and two levels of Scope were paired to six combinations. Each subject was exposed to two different diseases, one with Small and the other with Large Scope. The remaining factors were balanced within subjects. Each participant completed 480 trials in two sessions with two blocks of 120 trials, the first block of trials with a 3 s deadline and the second with a 1 s deadline. Note that within a given session, Disease and Scope conditions were the same. Participants had 5-min breaks between blocks and sessions.

The experimental trials started by showing the number of affected people for the corresponding trial. The subsequent screen showed the choice options (visualized by pie charts) and time limit for that particular trial. A response had to be made within the given time limit. The last screen provided feedback about the outcome of the choice. After offset of the screen, the next trial started (for screenshots and details see Supplementary material and Diederich et al., 2018). Participants filled the REI after the first session, the AOT before the second session, and the SIRI after the second session. Questions of each scale were presented in random order.

### 2.4. Data processing and statistical methods

For each instrument, we normalized the values recorded for the participants by subtracting the smallest measurable value of the instrument (*I*_*min*_) from the value recorded for each participant (*I*_*i*_) and divide the result by the highest measurable value of the instrument (*I*_*max*_) minus *I*_*min*_: $I_{norm}=\frac{I_{i}-Imin}{I_{max}-I_{min}}$.

We quantified the number of frame-inconsistent choices (FIC) of each participant by comparing the responses to gain-framed trials with those given to the identical counterpart in the loss frame. We counted a FIC when a participant's response to otherwise identical trials varied depending on the framing as gain or loss.

We first evaluated the data using descriptive statistics and Pearson correlations between the number of FIC and the normalized values of the instruments.

To analyze the effects of framing, choice problem characteristics, and individual differences on the proportion of choosing the gamble, we used generalized linear mixed models (GLMM; family: binomial, bound optimization: quadratic approximation) with random intercept variance across participants and sequence of stimuli presented (trial sequence). For the statistical analysis, we used the computing environment R (version 4.0.3; packages: “lme4,” “descr,” “Hmisc,” “psych,” “simr”; Bates et al., 2014; Green and MacLeod, 2016; Aquino, 2018; R Core Team, 2018; Revelle, 2020; Harrell, 2021).^2^

All models included the relative frequency of choosing the risky option as the dependent variable. Frame (Loss; Gain), Scope of affected people, with categories Small (basic values: 20, 40, 60, 80) and Large (100 times the Small values), Probabilities of surviving/dying (<0.5; >0.5), Disease (Infectious disease; Leukemia; AIDS), and Time (1 s; 3 s limit) were included as explanatory variables. The first categories served as references. Since the scores of some of the instruments are expected to be highly correlated with each other, a model including all instruments would be affected by the problem of multicollinearity. Therefore, we executed the model separately for each of the five instruments (main effects models), i.e., the sub-scales of the REI (RA and EX), the AOT, and the sub-scales of the SIRI (ST and IN). Furthermore, to investigate the relationship between a person's test score and the impact of the explanatory variables on risky choice, we included two-way interactions of the instrument scores by each explanatory variable in the models (interaction models).

A *post-hoc* sensitivity analysis indicating the smallest detectable effect sizes (using the R package “simr”; Green and MacLeod, 2016) is shown the Supplementary material (Supplementary Tables S1–S3).

### 2.5. Participants

Fifty-five undergraduates (26 female, 29 male) of Jacobs University Bremen participated in two experiment sessions (age: 18–26 years; median = 20; English speakers). Altogether, each participant performed 480 trials (384 test trials; 96 catch trials). The experiment lasted for about 90 min. See Diederich et al. (2018) for details.

### 2.6. Results

Of the 55 participants, 12 (7 females) have been excluded due to an unusually high number of catch trial failures (14 inferior responses in one block). Of the remaining 16,512 test trials (43 × 386), 80 trials were timeouts and were also removed from the data set. Thus, the following analysis is based on a total of 16,432 trials. In 51.1% of valid trials, the risky option was chosen. Overall, participants chose the risky option more often in loss trials (60.1%) than in gain trials (39.9%), indicating a framing effect (for details see Diederich et al., 2018). Probabilities and Scope had an impact on choice behavior: (1) The larger the probability of surviving/dying in the scenario was the higher the proportion of the risky choice option, and (2) the fewer people were affected (Scope: Small), the higher the proportion of the risky choice option (for details see Diederich et al., 2018).

#### 2.6.1. Individual differences in frame-inconsistent choices

The number of frame-inconsistent choices (FIC) ranged from 8 to 64 (overall: mean = 43.2, SD = 15.9) among the participants. That is, the average proportion of FIC was 67%. Note that the FIC proportions varied between the conditions of time limit (72% for 1 s, and 62% for 3 s time limit). The individual scores measured using the instruments varied across a moderate range. Details and normalized scores are found in the Supplementary material (Supplementary Table S4). We observed statistically significant correlations between scores of the following scales: ST and IN, ST and RA, ST and AOT, and IN and AOT. However, none of the instruments correlated significantly with FIC (see Table 1).

**Table 1:** Experiment 1: correlations between FIC and scores of risk-style (stimulating and instrumental risk) and cognitive-style (rational thinking, experiential thinking, and actively open-minded thinking style).

	**FIC**	**ST**	**IN**	**RA**	**EX**
ST	0.24				
*p*	*0.121*				
IN	0.16	**0.47**			
*p*	*0.315*	*0.002*			
RA	0.07	**0.43**	0.27		
*p*	*0.646*	*0.004*	*0.075*		
EX	−0.15	−0.27	0.02	−0.13	
*p*	*0.327*	*0.074*	*0.884*	*0.398*	
AOT	−0.19	**−0.31**	**−0.42**	−0.18	0.09
*p*	*0.214*	*0.046*	*0.005*	*0.257*	*0.552*

FIC, frame-inconsistent choice; ST, stimulating risk; IN, instrumental risk; RA, rational thinking; EX, experiential thinking; AOT, actively open-minded thinking. Statistically significant correlations (*p* < 0.05) are printed in bold and *p*-values are italicized.

#### 2.6.2. Individual differences in choice proportions

The main effects GLMM analyses (see Supplementary Tables S5–S9) showed no significant relationship between the scores measured using the instruments and proportions of choosing the gamble option. However, we found significant effects for Frame, Scope, and Probabilities but not for Disease and Time in each main effects model.

In the following, we report the results of the interaction effects GLMM analyses, separate for each instrument. Note that we interpret interactions even if the main effects were not significant. It is well possible that effects have canceled out due to the specific response behavior of participants with different risk-styles and cognitive-styles.

##### 2.6.2.1. Rational-experiential thinking

**Figure 1:**
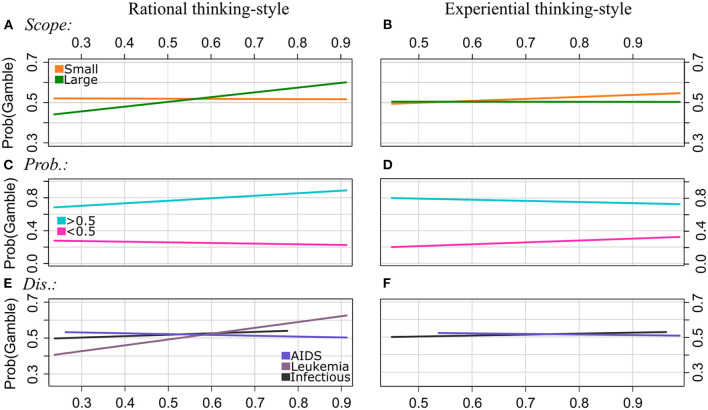
Experiment 1: Regression lines of the proportions of choosing the gamble option as a function of rational thinking-style (left column) and experiential thinking-style (right column), separately for the levels of Scope [patterns **(A, B)**], Probabilities **(C, D)**, and Disease **(E, F)**. Note that we applied a smaller range of values on the y-axis for the plots of the patterns **(A, B, D, E)** to illustrate the interaction effect more clearly.

Table 2 shows the results of the GLMM analyses and Figure 1 illustrates significant interaction effects. We interpret the findings as follows:

**Table 2:** Experiment 1: Generalized linear mixed models, Interactions: rational and experiential thinking-style.

**Rational thinking-style**				
**Fixed effects:**	**Est**.	**SE**	***z*** **-value**	***p*** **-value**
(Intercept)	0.215	0.552	0.389	0.697
RA	−1.609	1.056	−1.523	0.128
Frame (Gain)	−1.160	0.151	−7.705	<0.001
Scope (Large)	−0.611	0.168	−3.637	<0.001
Prob. (>0.5)	1.740	0.155	11.219	<0.001
Leukemia	−1.018	0.246	−4.132	<0.001
AIDS	−0.530	0.216	−2.455	0.014
Time (3 s)	−0.025	0.141	−0.179	0.858
RA × Frame	−0.395	0.294	−1.342	0.180
RA × Scope	1.117	0.316	3.537	<0.001
RA × Prob.	2.084	0.302	6.890	<0.001
RA × Leukemia	1.949	0.456	4.275	<0.001
RA × AIDS	0.895	0.420	2.131	0.033
RA × Time	0.160	0.273	0.586	0.558
**Random effects:**		**SD (Est.)**		
Trial seq. (Intercept)		0.024		
Subject (Intercept)		0.967		
**Experiential thinking-style**				
**Fixed effects:**	**Est**.	**SE**	***z*** **-value**	***p*** **-value**
(Intercept)	−1.747	0.932	−1.873	0.061
EX	1.697	1.300	1.306	0.192
Frame (Gain)	−1.427	0.253	−5.649	<0.001
Scope (Large)	0.589	0.272	2.168	0.030
Prob. (> 0.5)	3.670	0.260	14.114	<0.001
Leukemia	−0.001	0.370	−0.001	0.999
AIDS	1.017	0.325	3.128	0.002
Time(3 s)	0.077	0.237	0.324	0.746
EX × Frame	0.112	0.351	0.319	0.749
EX × Scope	−1.002	0.381	−2.629	0.009
EX × Prob.	−1.275	0.362	−3.519	<0.001
EX × Leukemia	−0.107	0.511	−0.210	0.834
EX × AIDS	−1.595	0.455	−3.508	<0.001
EX × Time	−0.033	0.330	−0.099	0.921
**Random effects:**		**SD (Est.)**		
Trial seq. (Intercept)		0.024		
Subject (Intercept)		0.967		

16,432 observations from *n* = 43 participants indicated in a series of 120 trials per block.

Frame: The GLMM analyses revealed no significant interaction effects of RA by Frame and EX by Frame. That is, the framing effect, i.e., divergence in proportions of choosing the gamble between gain and loss frames, was not moderated by rational or experiential thinking-style.

Scope: The effect of Scope on choosing the gamble was moderated by RA and EX. In particular, the proportions of gambling were lower for Scope Large than for Scope Small for individuals with lower RA scores (about <0.5). However, it increased with RA scores for Scope Large but not for Scope Small. The GLMM analysis of the EX scores showed that gambling increased with EX scores for Scope Small but not for Scope Large. That is, the effect of Scope reverses with increasing RA scores, and it becomes stronger with increasing EX scores.

Probabilities: Both RA and EX moderated the effect of Probabilities on choosing the gamble option. Participants chose the gamble more often for Probabilities >0.5, and they chose the sure option more often for Probabilities <0.5. Gambling proportions decreased with RA scores and increased with EX scores for Probabilities <0.5, and they increased with RA scores and decreased with EX scores for Probabilities >0.5. That is, the effect of Probabilities is getting stronger with increasing RA scores and it becomes weaker with increasing EX scores.

Disease: The effect of Disease types on the proportion of choosing the gamble option varied for individuals with different RA and EX scores, respectively. For Infectious disease, gambling increased with increasing RA and EX scores. For AIDS, however, it decreased with increasing RA and EX scores. Moreover, the GLMM revealed that gambling increased even stronger with RA scores for Leukemia than for Infectious disease.

Time: No significant interaction effects of RA by Time and EX by Time were observed.

##### 2.6.2.2. Actively open-minded thinking

**Figure 2:**
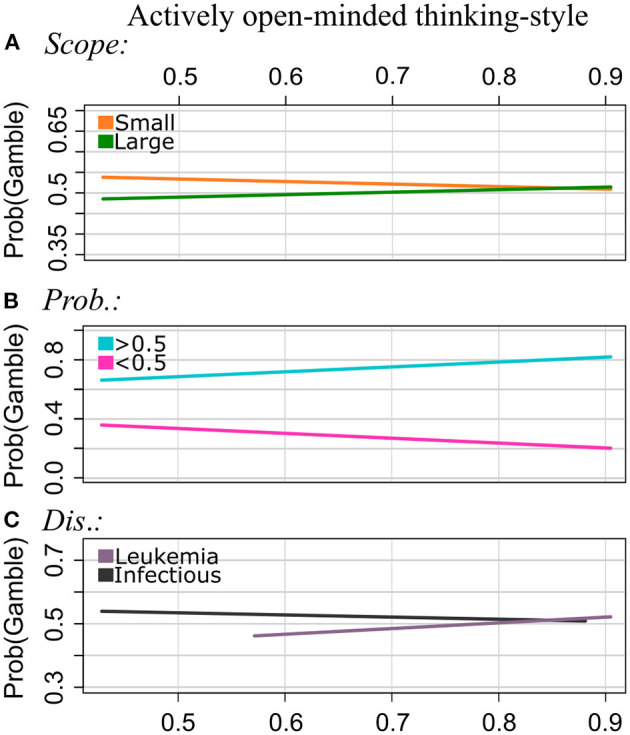
Experiment 1: Regression lines of the proportion of choosing the gamble option as a function of AOT, separately for the levels of Scope [pattern **(A)**], Probabilities **(B)**, and Disease **(C)**. Note that we applied a smaller range of values on the y-axis for the plots of the patterns **(A–C)** to illustrate the interaction effect more clearly.

Table 3 shows the interaction results when including AOT scores in the GLMM analysis. Figure 2 illustrates significant interactions.

**Table 3:** Experiment 1: Generalized linear mixed model, Interactions: actively open-minded thinking-style.

**Fixed effects:**	**Est**.	**SE**	***z*-value**	***p*-value**
(Intercept)	1.000	1.135	0.881	0.378
AOT	−2.213	1.531	−1.445	0.148
Frame (Gain)	−0.922	0.282	−3.274	0.001
Scope (Large)	−0.935	0.383	−2.440	0.015
Prob. (>0.5)	−0.754	0.288	−2.619	0.009
Leukemia	1.154	0.555	2.078	0.038
AIDS	−0.351	0.388	−0.906	0.365
Time (3 s)	0.092	0.268	0.341	0.733
AOT × Frame	−0.603	0.387	−1.559	0.119
AOT × Scope	1.200	0.521	2.302	0.021
AOT × Prob.	4.860	0.398	12.218	<0.001
AOT × Leukemia	−1.643	0.748	−2.196	0.028
AOT × AIDS	0.483	0.522	0.926	0.355
AOT × Time	−0.052	0.367	−0.143	0.887
**Random effects:**	**SD (Est.)**			
Trial seq. (Intercept)	<0.001	
Subject (Intercept)	1.014	

16,432 observations from *n* = 43 participants indicated in a series of 120 trials per block.

Frame: The GLMM showed no interaction effect of AOT by Frame. That is, AOT did not serve as a moderator of the framing effect in the current study.

Scope: Participants chose the gamble option more often for Scope Small than for Scope Large (main effect; see Supplementary Table S7). For Scope Small, the proportion of choosing the gamble decreased with increasing AOT scores, and for Scope Large it increased with increasing AOT scores. That is, the effect strength of Scope becomes smaller with increasing AOT scores.

Probabilities: For Probabilities >0.5, the risky option was chosen more often, whereas for Probabilities <0.5, the sure option was chosen more often. As for the other instruments, we found a significant interaction effect of AOT by Probabilities: Proportions of choosing the gamble decreased with increasing AOT scores for Probabilities <0.5, and they increased with AOT scores for Probabilities >0.5. As observed for rational thinking-style, the effect of Probabilities is getting stronger with increasing AOT scores.

Disease: Participants with normalized AOT scores around 0.5. and 0.6, which are lower AOT scores measured in the sample used for this study, chose the sure option more often for Leukemia and the gamble more often for the Infectious disease. The gambling frequency increased with AOT scores for Leukemia, and it decreased with increasing AOT scores for the Infections Disease.

Time: There were no significant interactions between AOT and Time.

##### 2.6.2.3. Stimulating-instrumental risk-style

**Figure 3:**
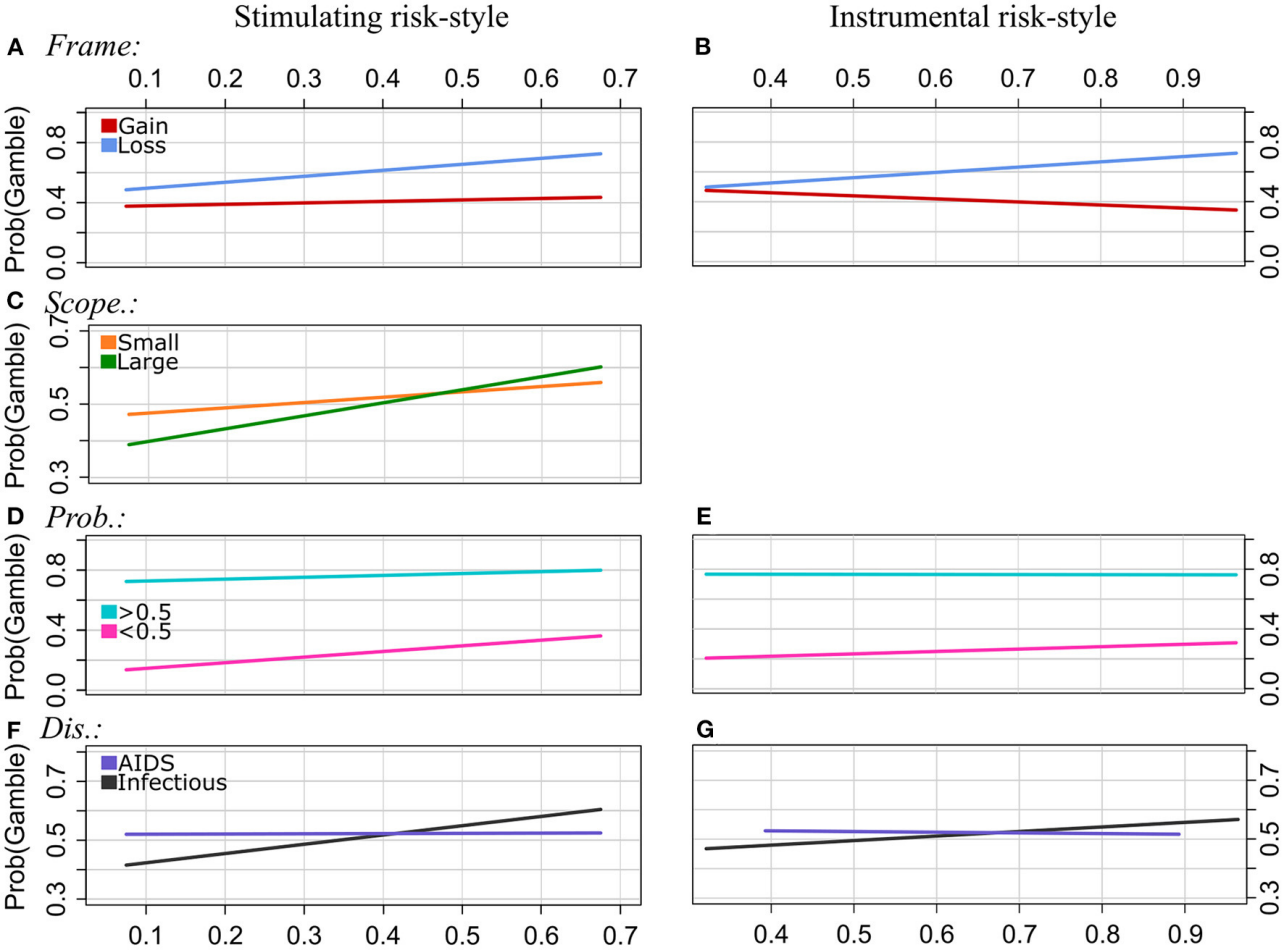
Experiment 1: Regression lines of the proportions of choosing the gamble option as a function of stimulating risk-style (left column) and instrumental risk-style (right column), separately for the levels of Frame [pattern **(A, B)**], Scope [**(C)**, effect of EX by Scope was n.s.], Probabilities **(D, E)**, and Disease **(F, G)**. Note that we applied a smaller range of values on the y-axis for the plots of the patterns **(C, F, G)** to illustrate the interaction effect more clearly.

Results of the GLMM analyses are shown in Table 4. Figure 3 displays significant interaction effects. We interpret the findings as follows:

**Table 4:** Experiment 1: Generalized linear mixed models, Interactions: stimulating and instrumental risk-style.

**Stimulating risk-style**				
**Fixed effects:**	**Est**.	**SE**	***z*** **-value**	***p*** **-value**
(Intercept)	−1.330	0.431	−3.087	0.002
ST	1.985	0.998	1.990	0.047
Frame (Gain)	−0.824	0.120	−6.885	<0.001
Scope (Large)	−0.814	0.134	−6.059	<0.001
Prob. (>0.5)	3.581	0.127	28.180	<0.001
Leukemia	−0.285	0.176	−1.619	0.105
AIDS	−0.636	0.161	−3.964	<0.001
Time (3 s)	0.021	0.114	0.185	0.853
ST × Frame	−1.285	0.274	−4.693	<0.001
ST × Scope	1.664	0.301	5.535	<0.001
ST × Prob.	−1.946	0.287	−6.777	<0.001
ST × Leukemia	0.497	0.409	1.213	0.225
ST × AIDS	1.175	0.352	3.339	<0.001
ST × Time	0.081	0.259	0.314	0.753
**Random effects:**		**SD (Est.)**		
Trial seq. (Intercept)		0.033		
Subject (Intercept)		0.979		
**Instrumental risk-style**				
**Fixed effects:**	**Est**.	**SE**	***z*** **-value**	***p*** **-value**
(Intercept)	−1.865	0.906	−2.058	0.040
IN	1.981	1.363	1.454	0.146
Frame (Gain)	0.412	0.246	1.676	0.094
Scope (Large)	−0.483	0.262	−1.844	0.065
Prob. (>0.5)	4.129	0.256	16.126	<0.001
Leukemia	−0.050	0.313	−0.161	0.872
AIDS	−0.847	0.375	−2.262	0.024
Time (3 s)	−0.332	0.234	−1.419	0.156
IN × Frame	−2.654	0.368	−7.214	<0.001
IN × Scope	0.564	0.390	1.448	0.148
IN × Prob.	−2.021	0.380	−5.317	<0.001
IN × Leukemia	−0.073	0.472	−0.155	0.877
IN × AIDS	1.118	0.548	2.037	0.042
IN × Time	0.583	0.348	1.676	0.094
**Random effects:**		**SD (Est.)**		
Trial seq. (Intercept)		0.026		
Subject (Intercept)		1.002		

16,432 observations from *n* = 43 participants indicated in a series of 120 trials per block.

Frame: The strength of the framing effect increases with scores of both stimulating risk-style and instrumental risk-style. In particular, the proportion of choosing the gamble option increases with ST scores in gain and loss frames. In loss frames, however, gambling proportions increase more strongly with ST scores than in gain frames. For the IN scale, there is a tendency of decreasing gambling proportions with increasing IN scores in the gain frame and increasing gambling proportions with increasing IN scores in the loss frame.

Scope: ST moderated the effect of Scope on risky choices. Overall, participants were less likely to choose the risky option for the Large Scope category than for Scope Small. However, the strength of the effect of Scope becomes smaller with increasing ST scores, and it even reverses at higher ST scores. The interaction effect of IN by Scope was not significant.

Probabilities: The gamble option was chosen more often for Probabilities >0.5, whereas, for Probabilities <0.5, the sure option was chosen more often. In the latter category (<0.5), the proportion of choosing the gamble increased with ST and IN scores. For high probabilities (>0.5), however, gambling frequency increased weaker or was relatively stable across participants with different scores of ST and IN, respectively. That is, the strength of the effect of Probabilities on choice behavior decreased with increasing scores of stimulating and instrumental risk-style.

Disease type: The GLMM analysis revealed a statistically significant interaction effect between the disease type “AIDS” and both risk-styles (ST and IN, respectively). For the reference category, i.e., “Infectious” disease, the proportion of choosing the gamble option tends to increase with increasing scores of ST and IN, respectively. However, for the “AIDS” disease category, gambling frequency was relatively stable across participants with different ST scores, and it slightly decreased with increasing IN scores.

Time: There were no significant interactions of ST by Time and IN by Time.

### 2.7. Summary and discussion

To investigate individual differences in susceptibility to risky choice framing, we used a psychophysical data collection approach and five different scales for measuring individual differences in cognitive-style and risk-style. We included two different interpretations of the framing effect. A narrow one, i.e., we compared the number of frame-inconsistent choices each participant made, and a wide one, i.e., we compared the proportions of choosing the gamble as a function of framing and other variables defining the choice problems. Overall, we found a high average proportion of frame-inconsistent choices (67%) and a strong effect of framing on the proportion of choosing the gamble (Gain: 40%; Loss: 60%).

The number of frame-inconsistent choices did not significantly correlate with the scores of any psychometric instrument used to measure cognitive-styles and risk-styles in the current study. Our findings are consistent with the results of Mandel and Kapler (2018), who investigated the impact of cognitive-styles (AOT and NFC) on the susceptibility to framing effects applying a narrow interpretation of frame-(in)consistent choices. In their between-subjects experiment, they counted a “frame-consistent choice” when a participant chose the sure option in the positive frame condition or the risky option in the negative frame condition. Neither AOT nor NFC correlated significantly with the number of frame-consistent choices.^3^ However, other studies showed statistically significant (*p* < 0.05) correlations between a measure of frame-(in)consistent choices and cognitive-styles (i.e., AOT, NFC; see e.g., Björklund and Bäckström, 2008; West et al., 2008; Peng et al., 2019; Erceg et al., 2022; Rachev et al., 2022), contradicting our results. According to the classification by Cohen (1988), these correlations are small to moderate. Furthermore, previous research on individual differences in framing susceptibility has paid most attention to measures of cognitive-style (Mandel and Kapler, 2018), and the relationship between risk-style and a narrow interpretation of the framing effect such as a measure of frame-(in)consistent choices has not been investigated so far.

For the wide framing effect interpretation, we found no impact of cognitive-style on framing effect strength which supports the majority of previous research applying a similar wide framing effect interpretation (Levin et al., 2002; Shiloh et al., 2002; LeBoeuf and Shafir, 2003; Björklund and Bäckström, 2008; Mahoney et al., 2011; Stark et al., 2017). Note that a few studies found a relationship between cognitive-style and framing effect strength in the wide interpretation. In particular, LeBoeuf and Shafir (2003), study 2 and Simon et al. (2004) found weaker framing effects for individuals with higher NFC scores.

In the current study, only stimulating risk-style and instrumental risk-style moderated the effect of framing on proportions of choosing the gamble. As expected, the framing effect becomes stronger as the scores of stimulating-risk increase. However, we observed the same pattern for instrumental risk-style, which is the opposite relationship than expected. High instrumental risk-style is theoretically associated with more deliberative risk-taking and, therefore, lower susceptibility to cognitive biases such as the framing effect (Zaleskiewicz, 2001). Mahoney et al. (2011), who also measured risk-style based on the intuitive-deliberative processing approach using the SIRI, found no significant moderator effects of stimulating risk-style and instrumental risk-style on framing effect strength. Note that the relationship between risk-style and framing effect strength has been investigated by previous studies using other concepts of risk-style (e.g., group polarization, risk-avoidance). The findings here are mixed (see Mahoney et al., 2011, for a review).

Moreover, we found that the effect of different outcomes (numbers of affected people; called Scope here) on risky choice behavior was moderated by rational thinking, experiential thinking, actively open-minded thinking, and stimulating risk-style. In line with the theory, we observed the effect of Scope to become stronger with increasing scores of experiential thinking-style, and it becomes weaker with increasing AOT scores. However, the other significant moderator effects were inconsistent with the basic assumptions of the scales. In particular, the effect of Scope reverses with increasing scores of rational thinking-style and stimulating risk-style. That is, individuals with lower scores chose the gamble less often, and those with higher scores chose the gamble more often for the large Scope than for the small one.

Each scale moderated the effects of probabilities of surviving/dying on choice behavior. Contrary to the theoretical implications, the effect of probabilities, i.e., selecting the sure option more often for probabilities <0.5 and the gamble more often for probabilities >0.5, becomes stronger with increasing scores of rational thinking-style and actively open-minded thinking-style, and it becomes weaker with increasing scores of experiential thinking-style. For the risk-style measures, we observed that the effect of probabilities becomes weaker with increasing scores. That is, only instrumental-risk moderated the effect of probabilities as expected.

The problem domain (different disease problems) was also moderated by each scale. In line with the theory, the effect of different disease problems on risky choice was found to become weaker with increasing AOT scores. However, the other measures of cognitive-style and risk-style moderated the effect of Disease in a different way than theoretically predicted. In particular, for the rational thinking-style, we expected that differences in the proportions of gambling between the three diseases will become smaller with increasing scores. For the experiential thinking-style, we expected to observe the opposite (i.e., differences in gambling proportion become larger with increasing scores). However, we observed that the frequency of choosing the gamble was lowest for Leukemia, higher for the Infectious disease, and highest for AIDS among individuals with the lowest scores of rational thinking-style. We observed the reversed order among individuals with the highest scores of rational thinking. Similarly, gambling proportions were lower for Leukemia than for AIDS among low experiential thinkers and lower for AIDS than for Leukemia among high experiential thinkers. A similar pattern emerged for the risk-style measures: For AIDS, the proportions of choosing the gamble was about the same (about 50%) for individuals with different scores of stimulating and instrumental risk-style. However, for the Infectious disease, individuals with low scores chose the sure option more often, and those with high scores chose the risky option more often.

We found no relationship between Time limits and any of the psychometric measures included in the current study.

Note that the current investigation is based on a reanalysis of existing data. However, the original study was not designed to measure correlations between frame-inconsistent choices and individual differences. Although the sensitivity analysis revealed a strong statistical power for the GLMM analyses, correlations require a much higher sample size to be stable (Schönbrodt and Perugini, 2013). Moreover, the lowest normalized scores of EX and AOT measured in the current study are 0.45, and 0.43 indicating a lack of participants with low and very low scores of these instruments. The small range of scores may be due to the sample size, which was determined for the analysis of particular effects in the original study, and the homogeneity of the sample. Using a student sample may result in a smaller range of scores for particular psychometric measures.

## 3. Experiment 2

Experiment 2 somewhat combines the social and psychophysical data collection approach. The experiment was conducted online. The composition of online samples might be more heterogeneous (e.g., age, education, profession) as compared to a student sample. We further increased the sample size to stabilize the correlations between frame-inconsistent choices and risk-style and cognitive-style, respectively. The general setup of the experiment was similar to the first one with a few exceptions described in the following. The study was conducted using Amazon MTurk and the online survey software EFS from TIVIAN. The statistical methods are the same as before.

### 3.1. Materials

We used the same three disease problem scenarios as in experiment 1 (unusual infectious disease, leukemia, and AIDS). As before, the Scope categories were Small and Large, with Small including only the values 20, 40, 60, and 80; for condition Scope Large, these numbers are multiplied by 100. The probabilities of surviving/dying were 0.3, 0.4, 0.6, and 0.7. For a given Scope, the 16 combinations (4 values × 4 probabilities) were framed as gains and losses, resulting in 32 test trials. In addition, 8 catch trials (4 per frame) were constructed to assess accuracy and engagement in the task. In four of the eight catch trials, participants were required to choose the sure option that offers a 100% chance to save all affected people. The risky option, however, involved a probability of 0.3 to save all people (no one will be saved with a probability of 0.7). In the other four catch trials, participants were required to choose the risky option that involved a probability of 0.7 to save all affected people. The sure option offered a 100% chance that no one will be saved. Based on pretesting, we allowed the participants to make two catch trial failures. The third catch trial failure led to the termination of the experiment.

One experimental block consisted of one of the three disease problem scenarios with 32 test trials and 8 catch trials. Different from experiment 1, we did not include different deadlines.

As in experiment 1, we used the same three measures (SIRI, REI, AOT). However, we replaced the 40-items REI with the shorter 10-items REI-short (Epstein et al., 1996). The reliability of the scales was α= 0.83 for the ST scale, α= 0.76 for the IN scale, α= 0.71 for the RA scale, α=0.84 for the EX scale, and α=0.81 for the AOT scale. Furthermore, we added attention checks to each scale (one to the AOT scale, and three to the REI and SIRI scale, respectively) where participants were asked to give a particular rating (e.g., “please rate this item with ‘4'”). An attention check failure terminated the experiment.

### 3.2. Design and procedure

The design was similar to the one used in experiment 1, however, with fewer trials per participant. Instead of completing 480 trials, each participant completed 80 trials in two blocks of 40 trials. Disease and Scope combinations varied between the two blocks. All trials had a response deadline of 5 s. Responses that were too slow (timeouts) were recorded as missing values and had no consequences for the participant. Timeouts in catch trails, however, were recorded as catch trial failures.

Upon inclusion in the study, participants first received basic information about the study. They were then introduced in the experimental procedure. The task was explained using two example trials (one per frame) with the components (e.g., choice options) labeled with explanatory comments. After participants remained for at least 60 s on the explanation page, they performed five practice trials. The first four practice trials included comments explaining the display. The first two practice trials had no response deadline. In practice trials 3 and 4, participants had to respond within the 5 s deadline. In case of a timeout, they were asked to repeat the corresponding practice trial. Practice trial 5 demonstrated how a test trial is displayed (i.e., explanatory comments disappeared).

Each block started with displaying the respective disease problem scenario. The procedure of the experimental trials and the display were similar to those in experiment 1 with the following modifications: (1) The screen displaying the number of affected patients was presented for 2 s (instead of 2.5 s). (2) The choice options were additionally labeled according to the frames (“patients survive” or “patients die,” respectively). There were no labels in experiment 1 (framing was only indicated by different gray shades). (3) The remaining time for a trial was indicated by a clock (instead of bars) counting down the seconds starting from 5 (screenshots and details can be found in the Supplementary material). (4) Participants were asked to use a standard computer mouse or an comparable input device for indicating their choice (instead of the left and right arrow-key of the keyboard).

Participants completed the AOT after the first block, the SIRI and the REI after the second block. Finally, they were asked for their age and gender. On the final page, participants received an individual, randomly generated code required to get the participation fee from MTurk.

### 3.3. Participants

We determined the sample size to match the valid observations in experiment 1 (384 test trials of 43 participants results in 16,512 test trials). The online experiment includes 64 test trials, which then require 258 participants. We requested participants on Amazon MTurk. They received a hyperlink that directed to the online experiment.

The online experiment was open for participation on Amazon MTurk from August 17^*th*^ to 19^*th*^, and on August 24^*th*^ 2021. MTurk workers were not required to meet any additional qualifications to participate (i.e., minimum HIT approval rate, language, location). On the fourth day, 1,327 workers accepted the HIT (human intelligence task) for participation. In total, 262 (117 female, 141 male, 4 preferred not to say) participants completed the experiment. The mean age was 32.73 years (median: 30, range: 20–64, SD: 9.64, *n* = 1 preferred not to say). Participants gave their informed consent prior to their inclusion in the study. The average completion time was 20 min and 27 s. Participants were paid a fixed amount of $4.70.

### 3.4. Results

Of the 1,327 individuals who accepted the HIT on MTurk for participation, 1,065 dropped out at the first pages showing the instructions, gave an incorrect response to an attention testing scale item, or failed more than two catch trials (see Supplementary material for an exploratory analysis of reasons for exclusion). We included data from the remaining 262 participants who finished the experiment. Of the 16,768 (262 × 64) test trials, 176 were timeouts and treated as missing values. Thus, the analysis is based on 16,592 trials. In 40.1% of the trials, the risky option was chosen. Overall, participants chose the risky option more often in loss trials (45.8%) than in gain trials (34.5%), indicating a framing effect. Furthermore, participants chose the risky option more often when Probabilities were large (>0.5: 47.1%) compared to small (<0.5: 33.1%). We found no effect of Scope and Disease on risky choice (see Table 5).

**Table 5:** Experiment 2: Main effects model.

**Fixed effects:**	**Est**.	**SE**	***z*-value**	***p*-value**
(Intercept)	−0.867	0.158	−5.498	<0.001
Frame (Gain)	−0.727	0.040	−18.212	<0.001
Scope (Large)	−0.025	0.040	−0.630	0.529
Prob. (>0.5)	0.900	0.056	22.454	<0.001
Leukemia	−0.065	0.056	−0.1.155	0.248
AIDS	−0.069	0.057	−1.210	0.226
**Random effects:**	**SD (Est.)**			
Subjects (Intercept)	2.377			
Trial seq. (Intercept)	0.027			

16,592 observations provided by *n* = 262 participants in a series of 40 trials per block.

#### 3.4.1. Individual differences in frame-inconsistent choices

Participants made between 0 and 32 frame-inconsistent choices (FIC) with a mean of 11.27 (SD = 9.99). That is, the mean proportion of FIC was 35%. Scores measured by the psychometric instruments varied across a wide range. Details and normalized values are found in the Supplementary material (Supplementary Table S4). We found no significant correlations between the instruments and the number of frame-inconsistent choices (see Table 6). However, we found a high number of significant correlations between the scores of the instruments: ST correlated positively with IN and EX, and it correlated negatively with RA and AOT. IN correlated positively with EX, and negatively with AOT. RA correlated positively with AOT, and negatively with EX. EX correlated negatively with AOT.

**Table 6:** Experiment 2: correlations between FIC and values of risk-style (stimulating and instrumental risk) and cognitive-style (rational thinking, experiential thinking, and actively open-minded thinking style).

	**FIC**	**ST**	**IN**	**RA**	**EX**
ST	0.08				
*p*	*0.225*				
IN	0.09	**0.69**			
*p*	*0.131*	* < 0.001*			
RA	0.01	**−0.20**	−0.05		
*p*	*0.874*	* < 0.001*	*0.380*		
EX	0.05	**0.51**	**0.52**	**−0.24**	
*p*	*0.367*	* < 0.001*	* < 0.001*	* < 0.001*	
AOT	−0.10	**−0.61**	**−0.40**	**0.52**	**−0.53**
*p*	*0.1210*	* < 0.001*	* < 0.001*	* < 0.001*	* < 0.001*

FIC, frame-inconsistent choice; ST, stimulating risk; IN, instrumental risk; RA, rational thinking; EX, experiential thinking; AOT, actively open-minded thinking. Statistically significant correlations (*p* < 0.05) are printed in bold and *p*-values are italicized.

#### 3.4.2. Individual differences in choice proportions

We found no impact of cognitive-styles and risk-styles on risky choices. The main effects models showed significant effects of Frame and Probabilities on the proportion of preferring the gamble over the sure option (see  Supplementary Tables S10–S14). As before, we show all interactions, separate for each instrument. Note that we interpret significant interaction effects even when the main effects were not significant. It is well possible that effects have been canceled out due to the specific response behavior depending on individual cognitive-style or risk-style.

##### 3.4.2.1. Rational-experiential thinking

**Figure 4:**
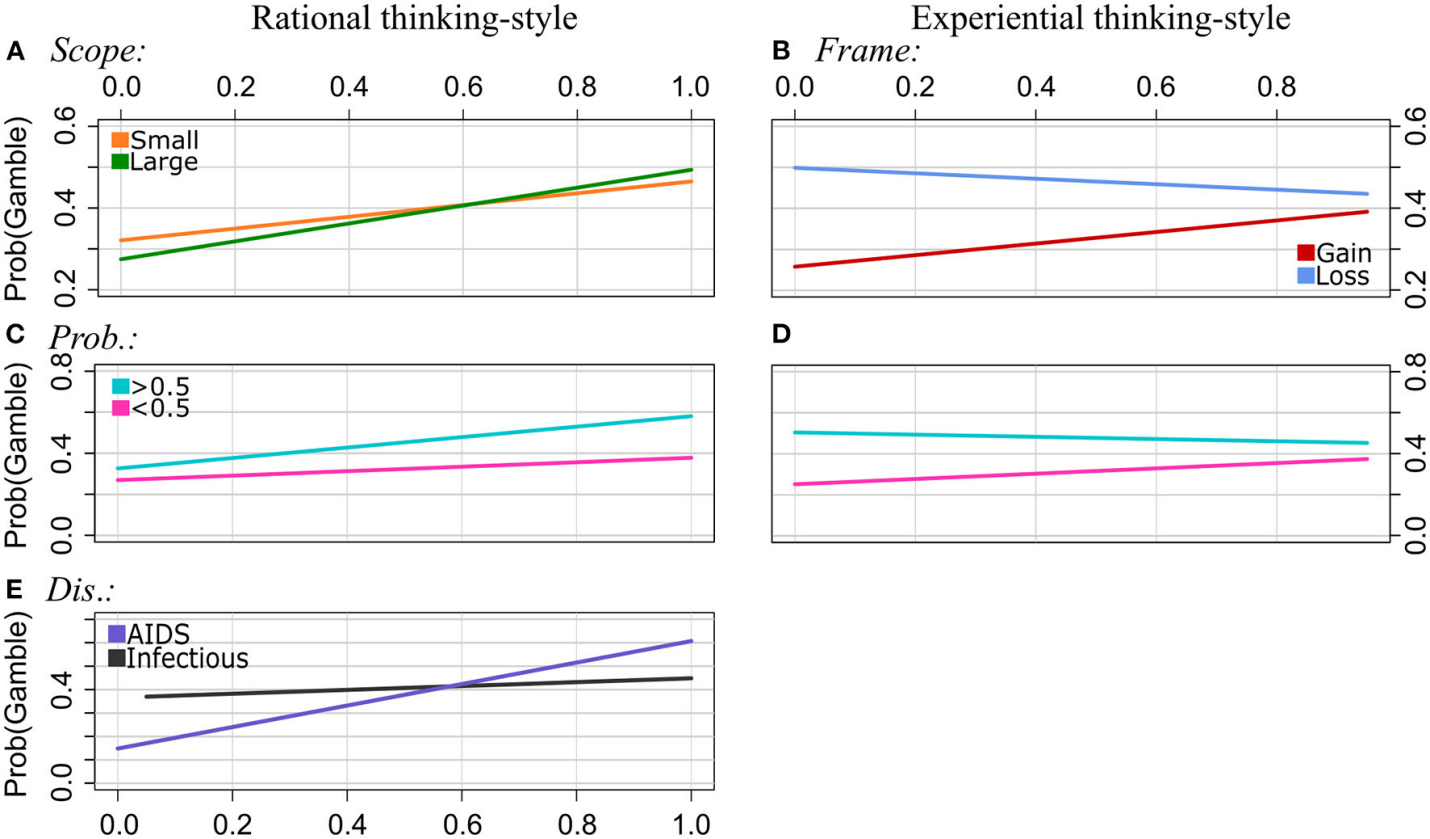
Experiment 2: Regression lines of the proportions of choosing the gamble option as a function of rational thinking-style (left column) and experiential thinking-style (right column), separately for the levels of Scope [pattern **(A)**, interaction effects of EX by Scope was n.s.], Frame [**(B)**, interaction effects of RA by Frame was n.s.], Probabilities **(C, D)**, and Disease [**(E)**, interaction effect of EX by Disease was n.s.]. Note that we applied a smaller range of values on the y-axis for the plots of the patterns **(A, B, E)** to illustrate the interaction effects more clearly.

The interaction effect analysis of rational and experiential thinking-styles revealed significant effects for EX by Frame, RA and EX by Scope, RA and EX by Probabilities, and RA by Disease. Table 7 shows the results and Figure 4 illustrates significant interaction effects. We interpret the results as follows:

**Table 7:** Experiment 2: Generalized linear mixed models, Interactions: rational and experiential thinking-style.

**Rational thinking-style**				
**Fixed effects:**	**Est**.	**SE**	***z*** **-value**	***p*** **-value**
(Intercept)	−1.046	0.507	−2.065	0.039
RA	0.336	0.843	0.398	0.691
Frame (Gain)	−0.695	0.130	−5.366	<0.001
Scope (Large)	−0.389	0.131	−2.970	0.003
Prob. (>0.5)	0.498	0.130	3.827	<0.001
Leukemia	−0.128	0.182	−0.702	0.483
AIDS	−0.774	0.188	−4.109	<0.001
RA × Frame	−0.059	0.214	−0.277	0.782
RA × Scope	0.647	0.217	2.976	0.003
RA × Prob.	0.699	0.216	3.240	0.001
RA × Leukemia	0.089	0.296	0.301	0.763
RA × AIDS	1.246	0.311	4.005	<0.001
**Random effects:**		**SD (Est.)**		
Subject (Intercept)		2.354		
Trial seq. (Intercept)		0.029		
**Experiential thinking-style**				
**Fixed effects:**	**Est**.	**SE**	***z*** **-value**	***p*** **-value**
(Intercept)	−0.986	0.556	−1.774	0.076
EX	0.183	0.863	0.212	0.832
Frame (Gain)	−1.561	0.143	−10.919	<0.001
Scope (Large)	0.099	0.141	0.702	0.483
Prob. (>0.5)	1.635	0.144	11.381	<0.001
Leukemia	−0.002	0.196	−0.013	0.990
AIDS	0.144	0.209	0.691	0.489
EX × Frame	1.340	0.220	6.104	<0.001
EX × Scope	−0.202	0.217	−0.933	0.351
EX × Prob.	−1.177	0.221	−5.339	<0.001
EX × Leukemia	−0.096	0.297	−0.323	0.747
EX × AIDS	−0.345	0.326	−1.057	0.291
**Random effects:**		**SD (Est.)**		
Subject (Intercept)		2.379		
Trial seq. (Intercept)		0.021		

16,592 observations provided by *n* = 262 participants in a series of 40 trials per block.

Frame: We found a significant interaction effect between EX and Frame. The frequency of choosing the risky option decreased with increasing EX scores in loss frames, and it increased with EX scores in gain frames. That is, the strength of the framing effect decreases with increasing EX scores. The interaction of RA by Frame was not statistically significant. Scope: The effect of Scope on choosing the gamble option was moderated by RA. In particular, gambling increased with higher RA scores in both categories of Scope. However, it increased stronger for Large than for Small Scope. EX did not moderate the effect of Scope.

Probabilities: Both RA and EX moderated the effect of Probabilities on choosing the risky option. Gambling proportions increased with RA scores and EX scores for Probabilities <0.5, and they increased even stronger with RA scores and decreased with EX scores for Probabilities >0.5. That is, the effect of Probabilities is getting stronger with increasing RA scores, and it becomes weaker with increasing EX scores.

Disease: We found a significant interaction effect between the RA scale and Disease. Specifically, the choice pattern for the Infectious disease was differed from that for AIDS. The gambling frequency increased with RA scores for both diseases. However, it increased even stronger with RA for AIDS than for the Infectious disease. No significant interaction effects between EX and the diseases were found.

##### 3.4.2.2. Actively open-minded thinking

**Figure 5:**
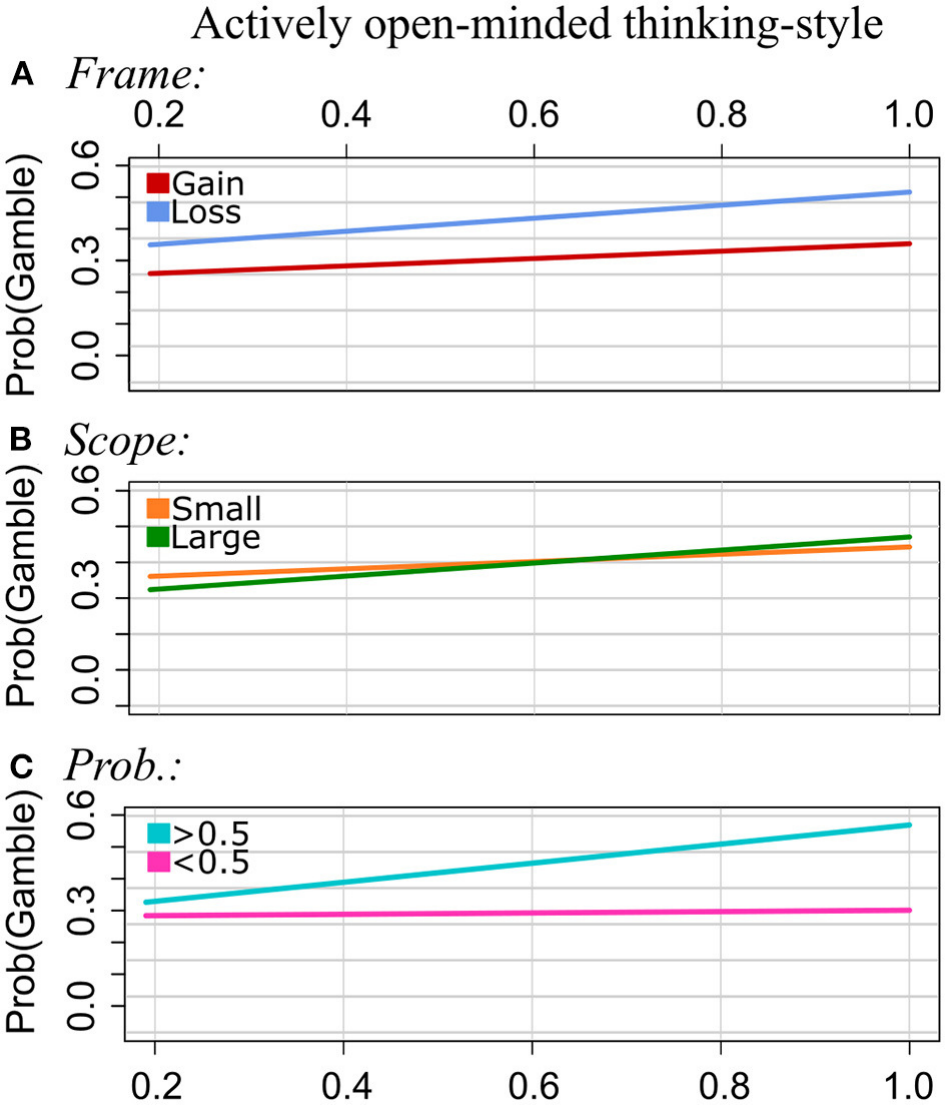
Experiment 2: Regression lines of the proportion of choosing the gamble option as a function of AOT, separately for the levels of Frame [pattern **(A)**], Scope **(B)**, and Probabilities **(C)**.

Table 8 shows the results of the interaction effects model when including actively open-minded thinking scores. We found significant interaction effects for AOT by Frame, AOT by Scope, and AOT by Probabilities. Significant effects are illustrated in Figure 5.

**Table 8:** Experiment 2: Generalized linear mixed model, Interactions: actively open-minded thinking-style.

**Fixed effects:**	**Est**.	**SE**	***z*-value**	***p*-value**
(Intercept)	−1.215	0.545	−2.231	0.026
AOT	0.569	0.858	0.664	0.507
Frame (Gain)	−0.436	0.141	−3.104	0.002
Scope (Large)	−0.332	0.140	−2.371	0.018
Prob. (> 0.5)	−0.017	0.141	−0.120	0.905
Leukemia	−0.152	0.194	−0.785	0.432
AIDS	0.002	0.203	0.008	0.993
AOT × Frame	−0.483	0.222	−2.174	0.030
AOT × Scope	0.508	0.220	2.305	0.021
AOT × Prob.	1.509	0.225	6.719	<0.001
AOT × Leukemia	0.155	0.309	0.501	0.616
AOT × AIDS	−0.114	0.317	−0.359	0.719
**Random effects:**	**SD (Est)**			
Subjects (Intercept)	2.369		
Trial seq. (Intercept)	0.025		

16,592 observations provided by *n* = 262 participants in a series of 40 trials per block.

Frame: The higher the AOT scores, the more often the gamble was chosen in gain frames and in loss frames. However, the increase was steeper in the loss condition. That is, the framing effect becomes stronger with increasing AOT values.

Scope: The proportions of choosing the gamble option increased with increasing AOT scores in both categories of Scope. However, this increase was stronger for Scope Large than for Scope Small.

Probabilities: Gambling strongly increased with AOT scores for Probabilities (>0.5). This effect was much weaker for Probabilities (<0.5). That is, the effect of Probabilities on risky choices becomes stronger with increasing AOT scores.

No interactions of AOT by Disease were found.

##### 3.4.2.3. Stimulating Instrumental Risk Inventory (SIRI)

The results of the interaction effect models show that both stimulating and instrumental risk-style moderate the effects of Frame and Probabilities. Based on the statistical significance shown in Table 9, we interpret the interaction effect as follows:

**Table 9:** Experiment 2: Generalized linear mixed models, Interactions: stimulating and instrumental risk-style.

**Stimulating risk-style**				
**Fixed effects:**	**Est**.	**SE**	***z*** **-value**	***p*** **-value**
(Intercept)	−1.101	0.435	−2.530	0.011
ST	0.493	0.895	0.551	0.582
Frame (Gain)	−1.356	0.116	−11.690	<0.001
Scope (Large)	0.030	0.114	0.262	0.794
Prob. (>0.5)	1.579	0.117	13.486	<0.001
Leukemia	0.106	0.158	0.671	0.502
AIDS	−0.205	0.174	−1.177	0.239
ST × Frame	1.366	0.235	5.801	<0.001
ST × Scope	−0.108	0.233	−0.463	0.644
ST × Prob.	−1.472	0.237	−6.206	<0.001
ST × Leukemia	−0.374	0.321	−1.165	0.244
ST × AIDS	0.293	0.357	0.821	0.412
**Random effects:**		**SD (Est.)**		
Subject (Intercept)		2.385		
Trial seq. (Intercept)		0.023		
**Instrumental risk-style**				
**Fixed effects:**	**Est**.	**SE**	***z*** **-value**	***p*** **-value**
(Intercept)	−0.778	0.654	−1.190	0.234
IN	−0.166	1.029	−0.161	0.872
Frame (Gain)	−2.416	0.179	−13.502	<0.001
Scope (Large)	−0.033	0.175	−0.189	0.850
Prob. (>0.5)	1.815	0.178	10.170	<0.001
Leukemia	0.091	0.237	0.383	0.702
AIDS	−0.485	0.281	−1.730	0.084
IN × Frame	2.692	0.276	9.745	<0.001
IN × Scope	0.028	0.272	0.104	0.917
IN × Prob.	−1.450	0.276	−5.257	<0.001
IN × Leukemia	−0.266	0.371	−0.718	0.473
IN × AIDS	0.652	0.431	1.514	0.130
**Random effects:**		**SD (Est.)**		
Subject (Intercept)		2.409		
Trial seq. (Intercept)		0.022		

16,592 observations provided by *n* = 262 participants in a series of 40 trials per block.

**Figure 6:**
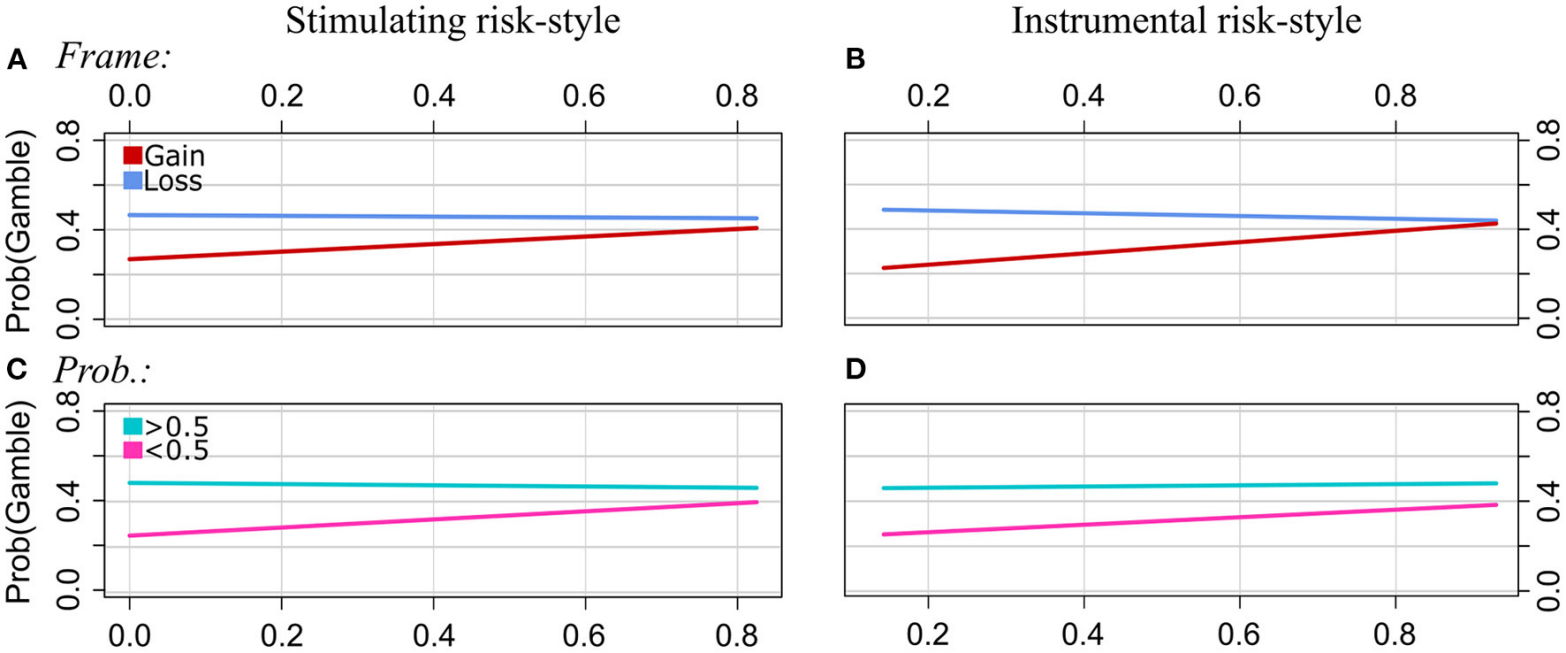
Experiment 2: Regression lines of the proportions of choosing the gamble option as a function of stimulating risk-style (left column) and instrumental risk-style (right column), separately for the levels of Frame [patterns **(A, B)**] and Probabilities **(C, D)**.

Frame: Participants chose the gamble less often in the gain frame than in the loss frame. As illustrated in Figure 6, gambling increased in the gain frame with ST and IN scores. In the loss frame, however, gambling proportions did not change substantially with ST scores, and they slightly decreased with increasing IN scores. The findings suggest that framing effects become weaker with increasing stimulating and instrumental risk-style.

Probabilities: For Probabilities (>0.5), the proportions of choosing the risky option were relatively stable for participants with different scores of ST and IN. However, gambling increased with IN and EX scores when probabilities were low (<0.5). That is, the strength of the effect of Probabilities on choice behavior decreased with increasing scores of stimulating and instrumental risk-style (Figure 6).

### 3.5. Summary and discussion

The risky choice framing of the choice options as gains and losses and the probabilities of surviving/dying influenced choice behavior: Participants chose the gamble option more often in the loss frame than in the gain frame, and they chose it more often for probabilities >0.5 than for probabilities <0.5. In contrast to experiment 1, no effect of the number of affected people (Scope) was found. That is, the social science approach involving psychophysical elements was able to replicate the effects of framing and different probabilities, but it failed to replicate the effect of Scope on choice behavior.

We found no significant correlations between our narrow framing effect interpretation, i.e., frame-inconsistent choices, and the scores measured using the psychometric instruments (REI, AOT, SIRI), supporting the results of experiment 1.

For the wide framing effect interpretation, i.e., the difference in proportions of choosing the gamble between the frames, we found that stimulating and instrumental risk-style, and experiential and actively open-minded thinking served as moderators of the framing effect in experiment 2. This finding is different from the results of experiment 1, where we found only stimulating and instrumental risk-style moderating the framing effect. In experiment 2, only instrumental risk-style moderated the framing effect as predicted by the theory. The other moderator effects have the opposite direction. In particular, the strength of the framing effect decreased with increasing scores of stimulating and instrumental risk-style. These are the opposite effects as observed in experiment 1. Moreover, the framing effect strength decreased with increasing scores of experiential thinking-style, and it increased with scores of actively open-minded thinking-style.

Furthermore, the results of experiment 2 show that, apart from the framing, other effects influencing the proportion of choosing the gamble option are moderated by cognitive-styles and risk-styles. As before, we found that some scales moderated the effects differently than one would expect from their underlying assumptions. In particular, the relationship between rational thinking-style and the effect of Scope was similar in both experiments. The effect of Scope reverses with increasing scores of rational thinking-style. Participants who scored low in rational thinking chose the gamble less often, and those scoring high chose the gamble more often for the Large than for Small Scope. Moreover, the same relationship was observed for the moderator effect of actively open-minded thinking-style on Scope in experiment 2. However, this was different from the finding in experiment 1, where the effect strength of Scope decreased with increasing scores of actively open-minded thinking. Note that, due to a higher heterogeneity of the sample composition, we observed a wider range of cognitive-style scores in experiment 2. Specifically, the sample of experiment 2 includes more participants with low scores of rational, experiential, and actively open-minded thinking. This finding might be an explanation for the failed replication of the main effect of Scope. It may simply have been canceled out due to the reversed effect direction for participants with low scores.

As in experiment 1, all measures of cognitive-style and risk-style moderated the effect of probabilities of surviving/dying for the (hypothetical) affected people. The findings replicate those of experiment 1. That is, only instrumental risk-style moderated the effect in a way that is in line with the theoretical assumptions. The other moderators show the opposite effect direction than expected according to the theory: The strength of the effect increased with scores of rational and actively open-minded thinking-style, and it decreased with increasing scores of experiential thinking-style and stimulating risk-style.

We found a relationship between disease problems and rational thinking-style in experiment 2. As compared to the Infectious Disease condition, gambling increased with scores of rational thinking for the AIDS problem. In experiment 1, however, we found that gambling decreased with the scores for the same scenario. Moreover, no other significant moderator effects of a scale on the effect of Disease on risky choice were found in experiment 2. This result is different from our findings in experiment 1, where we found that each of the scales moderated that particular effect in some way.

## 4. General discussion and conclusions

In the current study, we investigated the impact of individual intuitive and deliberative processing styles, i.e., rational, experiential, and actively open-minded thinking-style (Baron, 1993; Epstein, 1998), and stimulating and instrumental risk-styles (Zaleskiewicz, 2001), on the strength of risky choice framing effects. Previous research on that has shown mixed results, which might be explained by the large variety of methodological implementations. In particular, study designs varied (within vs. between-subject designs), framing effects have been interpreted in different ways, variables describing the decision problem beyond the framing of the choice options have been mostly ignored so far, and the studies often used student samples which are more homogeneous than, e.g., community or online samples.

We report two experiments involving elements of psychophysical data collection. For both experiments, we evaluated framing effects using two different interpretations: a narrow one, that is, we counted and compared the number of frame-inconsistent choices (FIC) participants made, and a wide interpretation, that is, we looked at the proportions of choosing the risky option depending on framing. Furthermore, our analysis considered other variables describing the decision problem, such as outcomes, probabilities, problem domains, and response time constraints. Experiment 1 was conducted in the lab using a student sample, and experiment 2 was conducted online using a more heterogeneous (with respect to e.g., age, education, and profession) online sample.

Once again, the psychophysical data collection approach has been shown to be an excellent method for measuring framing effects (see also, e.g., Guo et al., 2017; Diederich et al., 2020; Roberts et al., 2021; Wyszynski and Diederich, 2022). In addition to framing, we found other effects influencing the proportion of choosing the gamble. The findings of the strongest effects, i.e., framing and surviving/dying probability of the hypothetical people affected by a disease, could be replicated in experiment 2 using an approach that combined the social science approach with psychophysical elements. In both studies, participants chose the gamble more often in loss than in gain trials and for probabilities higher than 0.5 as compared to probabilities lower than 0.5. However, the less pronounced effect of Scope on choice behavior, i.e., fewer risky choices when more hypothetical people are affected by a disease, could not be replicated in our second experiment.

Results of both experiments consistently show no relationship between the number of FIC and cognitive-styles or risk-styles. The findings are in line with Mandel and Kapler (2018), who found no moderating effect of need for cognition (equivalent to rational thinking-style) and actively open-minded thinking on a similarly narrow interpretation of framing effects. However, other studies showed small (according to the classification of Cohen, 1988) but significant positive correlations suggesting the number of FIC to decrease with increasing scores of rational (LeBoeuf and Shafir, 2003; Björklund and Bäckström, 2008; Peng et al., 2019) and actively open-minded thinking-style (West et al., 2008; Erceg et al., 2022; Rachev et al., 2022). Note that the relationships between FIC (or a similar measure) and experiential thinking-style, and between FIC and stimulating-instrumental risk-styles have not been investigated so far. Taken together, our findings and those of previous research show, at best, a small effect of cognitive-styles and risk-styles based on the intuitive-deliberative processing approach on the number of FIC (or a similarly narrow interpretation of risky choice framing effects).

A different picture emerges when examining individual differences in cognitive-style and risk-style on framing effects in the wide interpretation, i.e., the impact of risky choice framing on the proportion of choosing the gamble: Although we did not find any impact of rational, experiential, and actively open-minded thinking-style on framing effect strength in experiment 1, experiment 2 showed that risky choice framing effects become weaker with increasing scores of experiential thinking-style and stronger with scores of actively open-minded thinking-style. According to the classification of Cohen (1988), the effect sizes are large and medium, respectively. Inconsistent with theoretical assumptions, the vast majority of previous research investigating rational-experiential thinking-style as moderator of the influence of framing on choice behavior have not found a direct relationship (see e.g., Levin et al., 2002; Shiloh et al., 2002; Björklund and Bäckström, 2008; Mahoney et al., 2011; Stark et al., 2017). Our findings on rational-thinking style support these studies. However, Simon et al. (2004) found a small (according to Cohen, 1988) moderator effect of rational thinking-style: in line with the theory, they observed stronger framing effects for individuals with lower need for cognition scores. Moreover, Mahoney et al. (2011) found in one of the five decision problems they used in their study that the strength of the framing effect increased with experiential thinking-style supporting the theory. In contrast, our second study revealed a strong relationship exhibiting the opposite direction (i.e., framing effect strength decreased with experiential thinking-style).

In contrast to previous research (Mahoney et al., 2011), we found in both experiments that stimulating and instrumental risk-style moderated the framing effect. However, the results of our two experiments are different: For both risk-styles, experiment 2 showed stronger framing effects for increasing scores; we found the opposite results (weaker framing effects with increasing scores) in experiment 1.

Furthermore, we found that the scores of the psychometric instruments we used to measure cognitive-styles and risk-styles moderated the effects of other problem-describing characteristics on risky choice. However, as for the framing effect, the directions of the effects, i.e, whether they were stronger or weaker for particular scale scores, were often different from what we expected according to the basic assumptions of the scales and also between our experiments. Some of the discrepancies could be explained by the more heterogeneous sample composition in experiment 2, where the scores of the instruments were measured on a broader range. It is also possible that other effects not considered in the current studies, as well as further interaction effects (e.g., 3-way-interactions), influence choice behavior. For instance, we know from previous studies that short time limits for making the risky choice enhance the framing effect (e.g., Guo et al., 2017; Diederich et al., 2018, 2020; Wyszynski and Diederich, 2022). In the current study, the effect of time limits was not moderated by scores of the psychometric instruments (see experiment 1), but it is well possible that individual cognitive-styles and risk-styles influence the relationship between time and framing or other interactions. However, the analysis of three-way interactions was not part of the current investigation, but they are worth being explored in future research. Moreover, it should also be questioned whether the scales actually measured precisely the individual differences they were supposed to measure.

## Data availability statement

The datasets presented in this study can be found in online repositories. The names of the repository/repositories and accession number(s) can be found below: Open Science Framework (https://osf.io/thgdz/).

## Ethics statement

The studies involving human participants were reviewed and approved by Jacobs University Ethics Committee. The patients/participants provided their written informed consent to participate in this study.

## Author contributions

MW: conceptualization, methodology, software, formal analysis, investigation, data curation, writing—original draft, writing—review and editing, visualization, and project administration. AD: conceptualization, methodology, validation, resources, writing—original draft, writing—review and editing, supervision, and funding acquisition. Both authors contributed to the article and approved the submitted version.

## Footnotes

^1^According to the World Health Organization best practices for the naming of new human infectious diseases, we use a more contemporary term without labeling the disease with a country or region of origin.

^2^Note that previous research often analyzed data with an ANOVA approach (e.g., Shiloh et al., 2002; LeBoeuf and Shafir, 2003; Mahoney et al., 2011). We use GLMMs since they have been shown to be more flexible, accurate, powerful, and suited for (categorical) data analysis (Kristensen and Hansen, 2004; Jaeger, 2008; Yu et al., 2022).

^3^Mandel and Kapler (2018) also measured numeracy and cognitive-ability in their study. They found, that only cognitive-ability (i.e., the Cognitive Reflection Task; Frederick, 2005) correlated with the number frame consistent choices.
