# Supplementary material for: Individual differences moderate effects in an Unusual Disease paradigm: A psychophysical data collection lab approach and an online experiment
Source: Front Psychol. 2023 Mar 28;14:1086699. doi: 10.3389/fpsyg.2023.1086699 (PMC10086346; doi:10.3389/fpsyg.2023.1086699)
Supplement: Supplementary file 1 [file Data_Sheet_1.pdf]

Supplementary Material

1 DISEASE-SCENARIOS

In both experiments, we used three different versions (scenarios) of Tversky and Kahneman's Unusual Disease Problem. For the control condition of the disease variable, the scenario described an outbreak of an unusual infectious disease. The other two scenarios were about a new agent to treat leukemia and a new agent to treat AIDS. The scenarios read as follows:

"Unusual infectious disease": "Imagine that the German government is preparing for the outbreak of an unusual infectious disease, which is expected to kill many people. Two alternative programs to combat the disease have been proposed. Both programs have different consequences for different groups of people. Assume that the exact scientific estimates of the consequences of the programs are as described in each scenario".

A new agent to treat leukemia: "Imagine that scientists found a new agent to treat leukemia. Every year, leukemia kills many people. Two alternative substances to combat leukemia have been developed. Both substances can cause serious side effects that lead to death. Some groups of persons are more affected by the side effects than others. Assume that the exact scientific estimates of the consequences of the substances are as described in each scenario".

A new agent to treat AIDS: "Imagine that scientists found a new agent to treat AIDS. Every year, AIDS kills many people. Two alternative substances to combat AIDS have been developed. Both substances can cause serious side effects that lead to death. Some groups of persons are more affected by the side effects than others. Assume that the exact scientific estimates of the consequences of the substances on the different groups of people are as described in each scenario".

2 QUESTIONAIRES

We measured cognitive-styles with two psychometric measures: 1.) The Rational-Experiential Inventory, including the rational-analytic (RA) and the experiential-intuitive (EX) sub-scales (Pacini and Epstein, 1999). Note that we used the 40-items REI in experiment 1 and the 10-items REI in experiment 2. And 2.) the 7-item short form of the Actively Open-Minded Thinking (AOT-7) scale as used in Haran et al. (2013).

We measured risk-styles with the Stimulating-Instrumental Risk Inventory (SIRI; Zaleskiewicz, 2001), including the stimulating-risk sub-scale (ST) and the instrumental-risk sub-scale (IN).

2.1 REI (experiment 1)

Rationality scale

– I try to avoid situations that require thinking in depth about something (−) [REI-10]
– I'm not that good at figuring out complicated problems (−)
– I enjoy intellectual challenges
– I am not very good at solving problems that require careful logical analysis (−)
– I don't like to have to do a lot of thinking (−) [REI-10]
– I enjoy solving problems that require hard thinking
– Thinking is not my idea of an enjoyable activity (−)
– I am not a very analytical thinker (−)

– Reasoning things out carefully is not one of my strong points (−)
– I prefer complex problems to simple problems
– Thinking hard and for a long time about something gives me little satisfaction (−)
– I don't reason well under pressure (−)
– I am much better at figuring things out logically than most people
– I have a logical mind
– I enjoy thinking in abstract terms
– I have no problem thinking things through carefully
– Using logic usually works well for me in figuring out problems in my life
– Knowing the answer without having to understand the reasoning behind it is good enough for me (−)
– I usually have clear, explainable reasons for my decisions
– Learning new ways to think would be very appealing to me

Experientiality scale
– I like to rely on my intuitive impressions
– I don't have a very good sense of intuition (−)
– Using my gut feelings usually works well for me in figuring out problems in my life
– I believe in trusting my hunches
– Intuition can be a very useful way to solve problems
– I often go by my instincts when deciding on a course of action
– I trust my initial feelings about people
– When it comes to trusting people, I can usually rely on my gut feelings
– If I were to rely on my gut feelings, I would often make mistakes (−)
– I don't like situations in which I have to rely on intuition (−)
– I think there are times when one should rely on one's intuition
– I think it is foolish to make important decisions based on feelings (−)
– I don't think it is a good idea to rely on one's intuition for important decisions (−)
– I generally don't depend on my feelings to help me make decisions (−)
– I hardly ever go wrong when I listen to my deepest gut feelings to find an answer
– I would not want to depend on anyone who described himself or herself as intuitive (−)
– My snap judgments are probably not as good as most people's
– I tend to use my heart as a guide for my actions
– I can usually feel when a person is right or wrong, even if I can't explain how I know
– I suspect my hunches are inaccurate as often as they are accurate (−)
A minus sign (−) denotes reverse scoring.

2.2 REI-short (experiment 2)

– I don't like to have to do a lot of thinking (−)
– I try to avoid situations that require thinking in depth about something (−)
– I prefer to do something that challenges my thinking abilities rather than something that requires little thought
– I prefer complex to simple problems
– Thinking hard and for a long time about something gives me little satisfaction (−)
– I trust my initial feelings about people
– I believe in trusting my hunches
– My initial impressions of people are almost always right

– When it comes to trusting people, I can usually rely on my “gut feelings”
– I can usually feel when a person is right or wrong even if I can’t explain how I know
A minus sign (−) denotes reverse scoring.

2.3 AOT

– Allowing oneself to be convinced by an opposing argument is a sign of good character
– People should take into consideration evidence that goes against their beliefs
– People should revise their beliefs in response to new information or evidence
– Changing your mind is a sign of weakness (−)
– Intuition is the best guide in making decisions (−)
– It is important to persevere in your beliefs even when evidence is brought to bear against them (−)
– One should disregard evidence that conflicts with one’s established beliefs (−)
A minus sign (−) denotes reverse scoring.

2.4 SIRI

Stimulating risk scale
– If I play a game (e. g., cards) I prefer to play for money
– I enjoy risk-taking
– I often take Risk just for fun
– I takerisk only if it is absolutely necessary to achieve an important goal (−)
– I am attracted by different dangerous activities
– While taking risk I have a feeling of a very pleasant flutter
– I avoid activities whose results depend too much on chance (−)
– Gambling seems something very exciting to me
– In business one should take risk only if the situation can be controlled (−)
– I make risky decisions quickly without an unnecessary waste of time

Instrumental risk scale
– At work I would prefer a position with a high salary which could be lost to a stable position but with a lower salary
– To achieve something in life one has to take risks
– If there is a big chance to profit I take even very high risks
– To gain high profits in business one has to take high risks.
– If there was a big chance to multiply the capital I would invest my money even in the shares of a completely new and uncertain firm
– I willingly take responsibility in my work-place
– The skill of reasonable risk-taking is one of the most important managerial skills
A minus sign (−) denotes reverse scoring.

3 DISPLAY EXPERIMENT 1

Screenshots of a practice trial and the display showing the number of affected people, the choice alternatives, and the feedback for a sample trial in experiment 1.

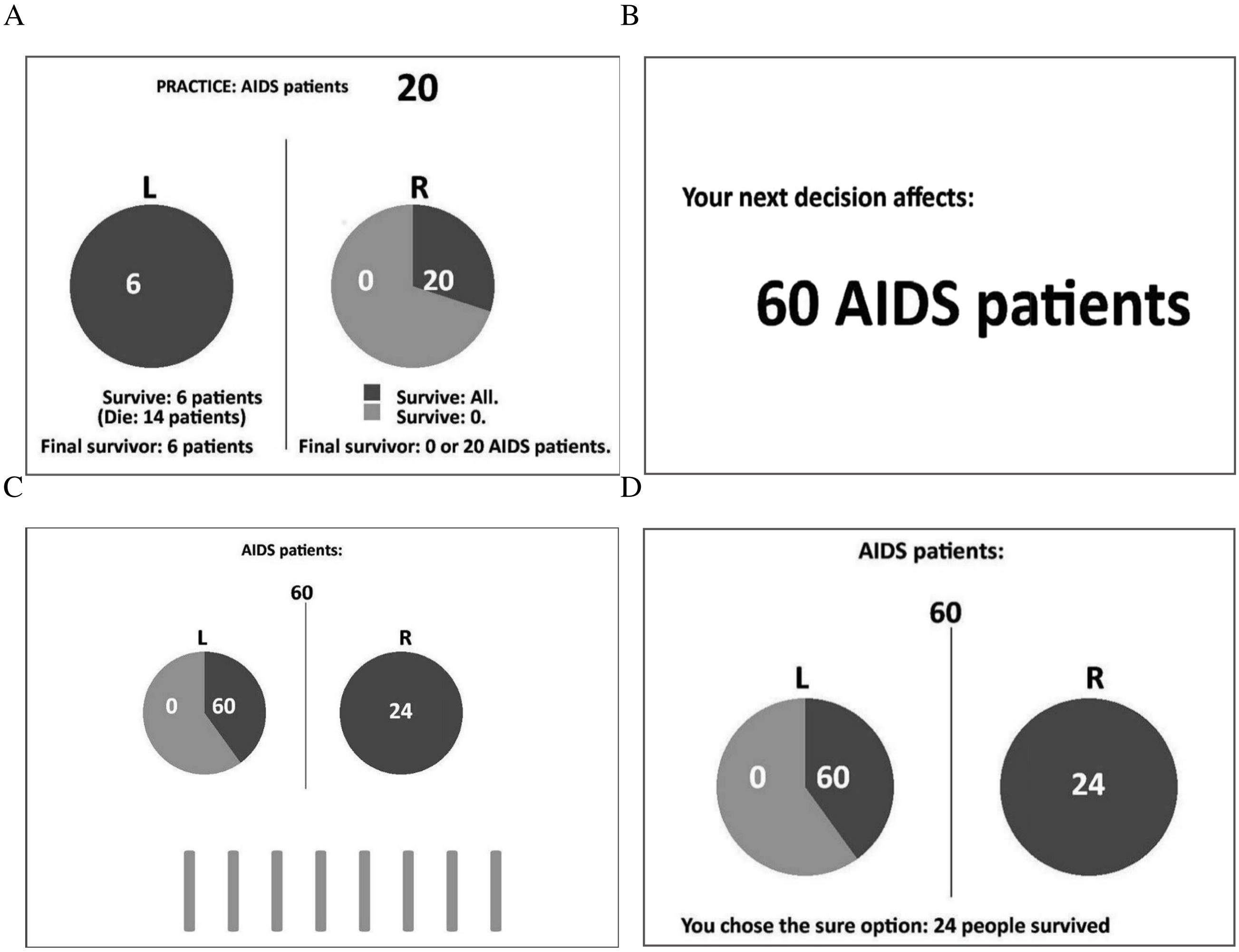

Figure S1. Experiment 1: Example of a guided practice trial (A) and timeline for one trial in a gain frame (B–D). The screen displaying the number of patients was presented for 2.5 sec (B). The screen displaying the choice was presented for either 1s or 3s, depending on the experimental condition (C). The bars below the pie-charts indicate the available time for particular trials (speed by which the bars were removed). The feedback screen (D) was presented for 2.5 sec, in which the result of the current trial was announced. The conditions in this sample are Frame=Gain; Disease=AIDS; Scope=Small; sure option was chosen.

4 DISPLAY EXPERIMENT 2

Screenshots of a practice trial and the display showing the number of affected people, the choice alternatives, and the feedback for a sample trial in experiment 2.

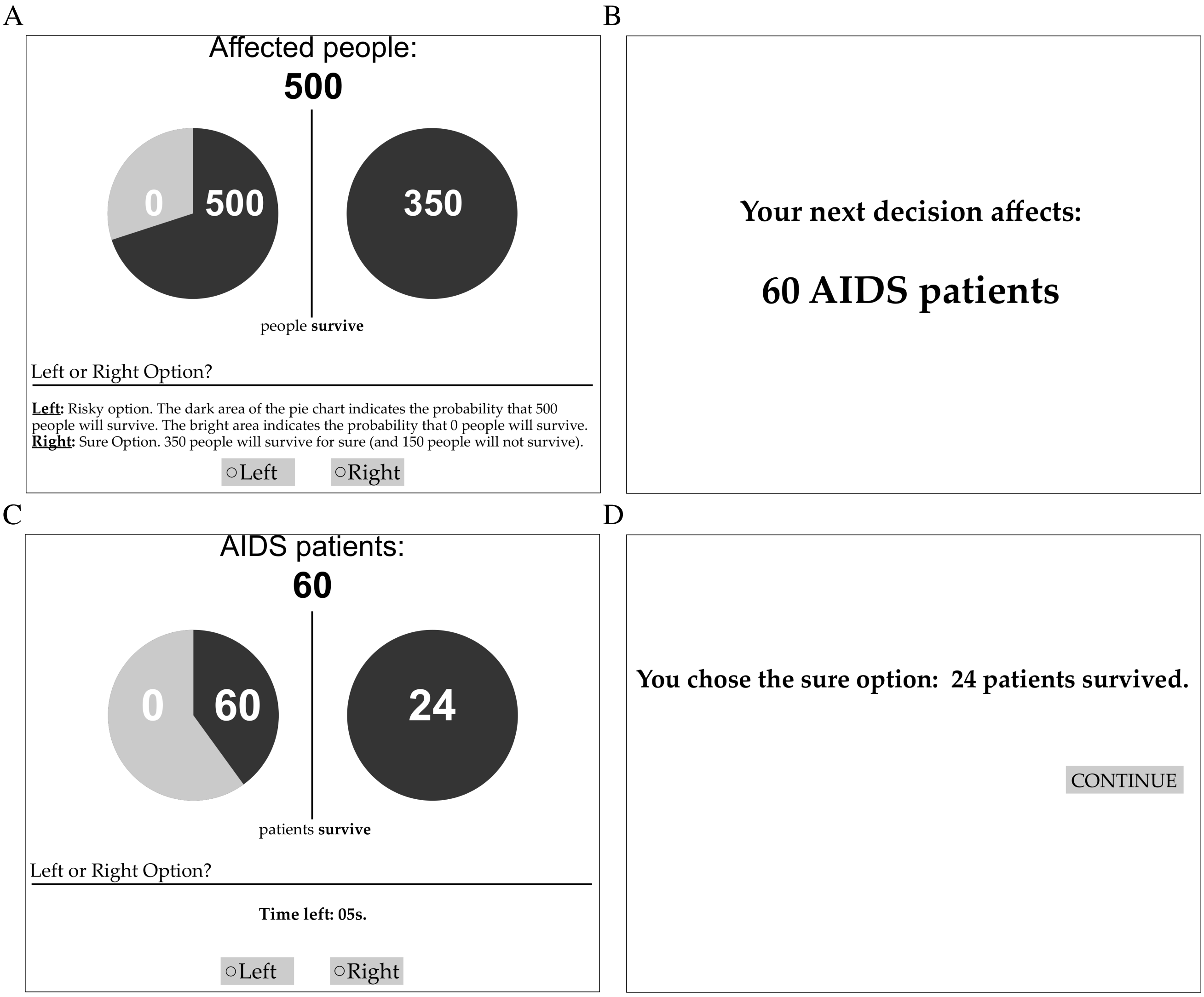

Figure S2. Experiment 2: Example of a practice trial (A) and timeline for one trial in a gain frame (B–D). The screen displaying the number of patients was presented for 2 sec (B). The screen displaying the choice was presented for 5s (C). The the number above the choice buttons indicate the available time for particular trials. The feedback screen (D) showed the result of the current trial was announced. The conditions in this sample are Frame=Gain; Disease=AIDS; Scope=Small; sure option was chosen.

5 EXPERIMENT 1: SENSITIVITY ANALYSIS OF INTERACTION EFFECTS

Results of experiment 1 based on a reanalysis of existing data (collected by Diederich et al., 2018). As an alternative to an a priori power analysis, we performed a post-hoc sensitivity analysis of the hypothesis-tests (GLMM, interaction effects models) to provide the minimum effect size needed to detect a real effect (i. e., effect actually existing) with a likelihood of 80%. Tables S3 – S2 show the smallest detectable effect size (at a statistical power of .8 and an α of .05) for each interaction effect tested with the interaction effects models. For comparison, the tables also show the estimated regression coefficients provided by the interaction models. Most non-significant effects have either very small sizes or are larger than the smallest detectable effect size indicating a sufficient ability of the models to detect the effect. The smallest detectable effect sizes were estimated using 300 Monte Carlo test simulation runs for each interaction effect. For simulations, we used the R package "simr" (Green and MacLeod, 2016).

Table S1. Experiment 1. Sensitivity analysis of interaction effects. Rational and Experiential thinking-style.

Rational thinking-style			Experiential thinking-style		
Interaction	Est.	Min. es	Interaction	Est.	Min. es
RA×Frame	−.395	−.864	EX×Frame	.112	.954
RA×Scope	1.117	.827	EX×Scope	−1.002	−1.066
RA×Prob.	2.084	.763	EX×Prob.	−1.275	−1.042
RA×Leukemia	1.949	1.116	EX×Leukemia	−.107	−1.456
RA×AIDS	.895	1.148	EX×AIDS	−1.595	−1.336
RA×Time	.160	.753	EX×Time	−.033	−.953

Note. Est.: Regression coefficients as provided by interaction models; Min. es: Smallest detectable effect size at a statistical power of .8 and an α of .05.

Table S2. Experiment 1. Sensitivity analysis of interaction effects. Actively open-minded thinking-style

Actively open-minded thinking-style		
Interaction	Est.	Min. es
AOT×Frame	−.603	−1.227
AOT×Scope	1.200	1.538
AOT×Prob.	4.860	1.033
AOT×Leukemia	−1.643	−2.112
AOT×AIDS	.483	1.498
AOT×Time	−.052	−1.045

Note. Est.: Regression coefficients as provided by interaction models; Min. es: Smallest detectable effect size at a statistical power of .8 and an α of .05.

Table S3. Experiment 1. Sensitivity analysis of interaction effects. Stimulating and Instrumental risk-style.

Stimulating risk-style			Instrumental risk-style		
Interaction	Est.	Min. es	Interaction	Est.	Min. es
ST×Frame	−1.285	−.775	IN×Frame	−2.654	−.907
ST×Scope	1.664	.796	IN×Scope	.564	1.069
ST×Prob.	−1.946	−.92	IN×Prob.	−2.021	−1.677
ST×Leukemia	.497	1.091	IN×Leukemia	−.073	−1.328
ST×AIDS	1.175	1.029	IN×AIDS	1.118	1.568
ST×Time	.081	.705	IN×Time	.583	1.073

Note. Est.: Regression coefficients as provided by interaction models; Min. es: Smallest detectable effect size at a statistical power of .8 and an α of .05.

6 VALUES OF RISK-STYLE AND COGNITIVE-STYLE MEASURED USING THE INSTRUMENTS

The individual scores measured using the psychometric instruments. Table S4 shows the range of raw and normalized scores. We normalized the scores by subtracting the smallest measurable value of the instrument (I_{min}) from the value recorded for each participant (I_i) and divide the result by the highest measurable value of the instrument (I_{max}) minus I_{min}: $I_{norm} = \frac{I_i - Imin}{I_{max} - I_{min}}$. Note that we used the 40-item REI in experiment 1, and a shorter version including 10-items in experiment 2.

Table S4. Values of risk-style and cognitive style: Rational thinking-style (RA), experiential thinking-style (EX), actively open-minded thinking-style (AOT), and stimulating risk-style (ST) and instrumental risk-style (IN).

Study	Instrument	Range raw	Range normalized
Exp. 1	RA	39 – 93 (11.99)	0.24 – 0.91 (0.15)
Exp. 2	RA	5 – 25 (3.71)	0 – 1 (0.19)
Exp. 1	EX	56 – 99 (9.75)	0.45 – 0.99 (0.12)
Exp. 2	EX	5 – 24 (3.64)	0 – 0.95 (0.18)
Exp. 1	AOT	25 – 45 (4.49)	0.43 – 0.9 (0.11)
Exp. 2	AOT	15 – 49 (7.71)	0.19 – 1 (0.18)
Exp. 1	ST	13 – 37 (6.41)	0.07 – 0.68 (0.16)
Exp. 2	ST	10 – 43 (7.11)	0 – 0.82 (0.18)
Exp. 1	IN	16 – 34 (3.35)	0.32 – 0.96 (0.12)
Exp. 2	IN	11 – 33 (4.42)	0.14 – 0.93 (0.16)

Note.. Standard deviations are shown in parentheses.

7 EXPERIMENT 1: REGRESSION MODELS, MAIN EFFECTS MODELS

To analyze the effects of framing, choice problem characteristics, and individual differences on the proportion of choosing the gamble, we used GLMM's with random intercept variance across participants and sequence of stimuli presented (trial sequence). Dependent variable: relative frequency of choosing the risky option. Explanatory variables: Frame (Loss; Gain), Scope of affected people, with categories Small (basic values: 20, 40, 60, 80) and Large (100 times the Small values), Probabilities of surviving/dying ($< .5$; $> .5$), Disease (Infectious disease; Leukemia; AIDS), and Time (1s; 3s limit). We executed the model separately for each of the five instruments.

Table S5. Experiment 1, GLMM. Main effects: rational thinking-style.

Fixed effects:	Est.	SE	z-value	p-value
(Intercept)	−.956	.532	−1.797	.072
RA	.795	1.011	.787	.432
Frame(Gain)	−1.343	.043	−31.510	<.001
Scope(Large)	−.102	.041	−2.521	.012
Prob.($> .5$)	2.770	.044	62.373	<.001
Leukemia	−.084	.057	−1.474	.140
AIDS	−.087	.055	−1.576	.115
Time(3s)	.054	.040	1.340	.180
Random effects:	SD(Est.)			
Trial seq. (Intercept)	.016			
Subject (Intercept)	.973			

Note. Number of observations:16,432; 120 trials per block; n=43 participants.

Table S6. Experiment 1, GLMM. Main effects: experiential thinking-style.

Fixed effects:	Est.	SE	z-value	p-value
(Intercept)	−.632	.900	−.702	.482
EX	.107	1.253	.085	.932
Frame(Gain)	−1.343	.043	−31.510	<.001
Scope(Large)	−.102	.041	−2.522	.012
Prob.(> .5)	2.770	.044	62.373	<.001
Leukemia	−.084	.057	−1.474	.140
AIDS	−.087	.055	−1.579	.114
Time(3s)	.054	.040	1.340	.180
Random effects:	SD(Est.)			
Trial seq. (Intercept)	.016			
Subject (Intercept)	.980			

*Note.*Number of observations:16,432; 120 trials per block; n=43 participants.

Table S7. Experiment 1, GLMM. Main effects: actively open-minded thinking-style.

Fixed effects:	Est.	SE	z-value	p-value
(Intercept)	−.657	1.063	−.618	.537
AOT	.136	1.427	.095	.924
Frame(Gain)	−1.343	.043	−31.510	<.001
Scope(Large)	−.102	.041	−2.522	.012
Prob.(> .5)	2.770	.044	62.373	<.001
Leukemia	−.084	.057	−1.476	.140
AIDS	−.087	.055	−1.581	.114
Time(3s)	.054	.040	1.340	.180
Random effects:	SD(Est.)			
Trial seq. (Intercept)	.016			
Subject (Intercept)	.980			

Note. Number of observations:16,432; 120 trials per block; n=43 participants.

Table S8. Experiment 1, GLMM. Main effects: stimulating risk-style.

Fixed effects:	Est.	SE	z-value	p-value
(Intercept)	−1.258	.396	−3.179	.001
ST	1.758	.915	1.922	.055
Frame(Gain)	−1.343	.043	−31.510	<.001
Scope(Large)	−.102	.041	−2.523	.012
Prob.(> .5)	2.770	.044	62.372	<.001
Leukemia	−.083	.057	−1.462	.144
AIDS	−.087	.055	−1.576	.115
Time(3s)	.054	.040	1.340	.180
Random effects:	SD(Est.)			
Trial seq. (Intercept)	.016			
Subject (Intercept)	.940			

Note. Number of observations:16,432; 120 trials per block; n=43 participants.

Table S9. Experiment 1, GLMM. Main effects: instrumental risk-style.

Fixed effects:	Est.	SE	z-value	p-value
(Intercept)	−.854	.848	−1.008	.314
IN	.456	1.275	.357	.721
Frame(Gain)	−1.343	.043	−31.510	<.001
Scope(Large)	−.102	.041	−2.523	.012
Prob.(> .5)	2.770	.044	62.373	<.001
Leukemia	−.084	.057	−1.471	.141
AIDS	−.087	.055	−1.582	.114
Time(3s)	.054	.040	1.340	.180
Random effects:	SD(Est.)			
Trial seq. (Intercept)	.016			
Subject (Intercept)	.979			

Note. Number of observations:16,432; 120 trials per block; n=43 participants.

8 EXPERIMENT 2: EXPLORATORY ANALYSIS OF CATCH TRIAL FAILURES

Data from 1,065 participants were excluded from the analysis. Forty-three participants were excluded due to giving an incorrect response to one of the seven attention test items included in the psychometric measures (e. g., “this item is to assess your accuracy and engagement. Please rate this item with ‘4”’). One thousand twenty-two participants gave an inferior response to more than two catch trials which resulted in the exclusion. Eight hundred twenty-nine of the 1021 participants failed three catch trials within the first 40 trials (i .e., during the first block). From these 829, 364 showed monotonous choice patterns, i. e., they chose in at least 96% of trials always the left or always the right option, or they chose the left and right option alternating; 104 chose in at least 96% of all trials always the sure option or always the risky option. One hundred seventy-seven participants produced a high amount of timeouts (meaning at least 10% of all trials were timeouts; mean: 18%). Recall that timeouts in catch trials were counted as inferior response.

We further observed some irregularities in the time needed to read the first two pages (introduction and instructions) and perform the practice trials. Of the 1,022 catch trial-failing participants, 193 needed less than 5 seconds to read the text presented on the first two pages. Eight participants needed more than 15 minutes (900 seconds) to complete the five practice trials (the average time for finishing the practice for the remaining participants was 226.32 seconds). One participant finished practice after 598,214 seconds (approximately seven days). At least one participant tried to circumvent the time limit by repeatedly refreshing the browser window.

Although we found no explanation for some catch trial failures, most of them seem to occur due to a lack of attention, clicking through the trials, or performing the trials carelessly (e. g., many timeouts). Note that MTurk workers were not pre-selected by requiring additional qualifications such as a minimum HIT approval rate, particular language skills, or a location.

9 EXPERIMENT 2: REGRESSION MODELS, MAIN EFFECTS MODELS

To analyze the effects of framing, choice problem characteristics, and individual differences on the proportion of choosing the gamble, we used GLMM’s with random intercept variance across participants and sequence of stimuli presented (trial sequence). Dependent variable: relative frequency of choosing the risky option. Explanatory variables: Frame (Loss; Gain), Scope of affected people, with categories Small (basic values: 20, 40, 60, 80) and Large (100 times the Small values), Probabilities of surviving/dying

(< .5; > .5), and Disease (Infectious disease; Leukemia; AIDS). We executed the model separately for each of the five instruments.

Table S10. Experiment 2, GLMM. Main effects: rational thinking-style.

Fixed effects:	Est.	SE	z-value	p-value
(Intercept)	−1.674	.486	−3.441	<.001
RA	1.416	.807	1.755	.079
Frame(Gain)	−.727	.040	−18.212	<.001
Scope(Large)	−.025	.040	−.631	.528
Prob.(> .5)	.900	.040	22.454	<.001
Leukemia	−.065	.056	−1.146	.252
AIDS	−.068	.057	−1.193	.233
Random effects:	SD(Est.)			
Subject (Intercept)	2.360			
Trial seq. (Intercept)	.27			

Note. Number of observations: 16,592; n=262 participants; 40 trials per block.

Table S11. Experiment 2, GLMM. Main effects: experiential thinking-style.

Fixed effects:	Est.	SE	z-value	p-value
(Intercept)	−.801	.533	−1.502	.133
EX	−.106	.826	−.129	.897
Frame(Gain)	−.727	.040	−18.212	<.001
Scope(Large)	−.025	.040	−.630	.529
Prob.(> .5)	.900	.040	22.454	<.001
Leukemia	−.065	.056	−1.155	.248
AIDS	−.069	.057	−1.210	.226
Random effects:	SD(Est.)			
Subject (Intercept)	2.377			
Trial seq. (Intercept)	.27			

Note. Number of observations: 16,592; n=262 participants; 40 trials per block.

Table S12. Experiment 2, GLMM. Main effects: actively open-minded thinking-style.

Fixed effects:	Est.	SE	z-value	p-value
(Intercept)	−1.732	.521	−3.322	<.001
AOT	1.423	.817	1.742	.082
Frame(Gain)	−.727	.040	−18.212	<.001
Scope(Large)	−.025	.040	−.629	.529
Prob.(> .5)	.900	.040	22.453	<.001
Leukemia	−.066	.056	−1.163	.245
AIDS	−.069	.057	−1.218	.223
Random effects:	SD(Est.)			
Subject (Intercept)	2.363			
Trial seq. (Intercept)	.27			

Note. Number of observations: 16,592; n=262 participants, 40 trials per block.

Table S13. Experiment 2, GLMM. Main effects: stimulating risk-style.

Fixed effects:	Est.	SE	z-value	p-value
(Intercept)	−.956	.416	−2.302	.021
ST	.198	.849	.234	.815
Frame(Gain)	−.727	.040	−18.212	<.001
Scope(Large)	−.025	.040	−.630	.529
Prob.(> .5)	.900	.040	22.454	<.001
Leukemia	−.065	.056	−1.154	.248
AIDS	−.069	.057	−1.210	.226
Random effects:	SD(Est.)			
Subject (Intercept)	2.376			
Trial seq. (Intercept)	.27			

Note. Number of observations: 16,592; n=262 participants; 40 trials per block.

Table S14. Experiment 2, GLMM. Main effects: instrumental risk-style.

Fixed effects:	Est.	SE	z-value	p-value
(Intercept)	−1.024	.612	−1.672	.094
IN	.256	.963	.266	.790
Frame(Gain)	−.727	.040	−18.212	<.001
Scope(Large)	−.025	.040	−.630	.529
Prob.(> .5)	.900	.040	22.454	<.001
Leukemia	−.065	.056	−1.152	.249
AIDS	−.069	.057	−1.211	.226
Random effects:	SD(Est.)			
Subject (Intercept)	2.375			
Trial seq. (Intercept)	.27			

Note. Number of observations: 16,592; n=262 participants; 40 trials per block.

REFERENCES

Diederich, A., Wyszynski, M., and Ritov, I. (2018). Moderators of framing effect in variations of the Asian Disease problem: time constraint, need, and disease type. *Judgment and Decision Making* 13, 529–546

Green, P. and MacLeod, C. J. (2016). SIMR: an R package for power analysis of generalized linear mixed models by simulation. *Methods in Ecology and Evolution* 7, 493–498

Haran, U., Ritov, I., and Mellers, B. A. (2013). The role of actively open-minded thinking in information acquisition, accuracy, and calibration. *Judgment and Decision Making* 8, 188–201

Pacini, R. and Epstein, S. (1999). The relation of rational and experiential information processing styles to personality, basic beliefs, and the ratio-bias phenomenon. *Journal of Personality and Social Psychology* 76, 972–987

Zaleskiewicz, T. (2001). Beyond risk seeking and risk aversion: personality and the dual nature of economic risk taking. *European Journal of Personality* 15, S105–S122
